# IRG1/Itaconate Inhibits Microglial Senescence-Like Transition by Modulating Mitochondrial Dynamics through Rhoa Alkylation in Subarachnoid Hemorrhage

**DOI:** 10.14336/AD.2025.1527

**Published:** 2026-04-01

**Authors:** Zeyu Zhang, Yanlin Chen, Weizhen Gao, Fengchen Zhang, Yichao Jin, Jie Li, Jiaqi Wang, Tao Lv, Feng Jia, Xiaohua Zhang

**Affiliations:** ^1^Department of Neurosurgery, Renji Hospital, Shanghai Jiao Tong University School of Medicine, Shanghai, China.; ^2^Key Laboratory of Anesthesiology, Shanghai Jiao Tong University, Ministry of Education, Shanghai, China.

**Keywords:** subarachnoid hemorrhage, itaconate, microglial senescence, mitochondrial dynamics, RhoA, immunometabolism

## Abstract

Aging is a potent risk factor for poor prognosis in subarachnoid hemorrhage (SAH), yet the molecular mechanisms underlying the age-related exacerbation of early brain injury remain incompletely understood. This study investigates the immunometabolic regulation of the microglial senescence-like transition following SAH, focusing on the immune-responsive gene 1 (IRG1)/itaconate axis. We observed that the endogenous upregulation of IRG1 and itaconate is a protective response to hemorrhagic stress that is significantly blunted in aged mice. Microglia-specific IRG1 deficiency exacerbated SAH-induced brain injury, characterized by an accelerated senescence-like transition and the secretion of senescence-associated secretory phenotype (SASP) factors. Mechanistically, we demonstrate that IRG1 deficiency leads to excessive mitochondrial fission and dysfunction via the hyperactivity of Dynamin-related protein 1 (Drp1). Using click chemistry-based proteomics and site-directed mutagenesis, we identified that itaconate exerts its neuroprotective effects by directly alkylating the small GTPase RhoA at the cysteine 107 (C107) residue. This specific post-translational modification inhibits RhoA-GTP binding and downstream ROCK1 activation, thereby suppressing Drp1-mediated mitochondrial fragmentation. Importantly, treatment with the cell-permeable itaconate derivative 4-octyl itaconate (4-OI) rescued mitochondrial dynamics and attenuated microglial senescence and neurological deficits, whereas the RhoA-C107S mutation abolished these protective effects. Collectively, our findings unveil a novel metabolic-mitochondrial checkpoint involving the IRG1/itaconate-RhoA-Drp1 axis. Restoring this pathway represents a promising therapeutic strategy to combat the age-related exacerbation of neuroinflammation and improve outcomes in SAH patients.

## INTRODUCTION

Subarachnoid hemorrhage (SAH) remains a catastrophic cerebrovascular event associated with high mortality and severe long-term disability [1]. Despite advances in neurosurgical and critical care management, the prognosis for SAH patients, particularly the elderly, remains dismal [1-3]. A growing body of evidence suggests that early brain injury (EBI), which occurs within the first 72 hours following aneurysm rupture, is the primary determinant of poor clinical outcomes [4]. EBI is characterized by a complex cascade of pathological events, including blood-brain barrier disruption, oxidative stress, and profound neuroinflammation [4, 5]. Notably, epidemiological studies indicate that age is a potent independent risk factor for poor recovery after SAH [3, 6]. However, the specific molecular mechanisms by which aging exacerbates the neuroinflammatory response and aggravates EBI remain incompletely understood.

Microglia, the resident immune cells of the central nervous system (CNS), are first responders to hemorrhagic insults [7, 8]. While physiological microglial activation is essential for debris clearance, excessive or dysregulated activation contributes to secondary brain injury [9]. Recent studies have highlighted a novel pathological state known as the senescence-like transition in microglia under stress conditions [10-13]. This phenotype is characterized by cell cycle arrest and the secretion of a senescence-associated secretory phenotype (SASP) factors, including pro-inflammatory cytokines (e.g., IL-1β, IL-6) and chemokines, which perpetuate a chronic inflammatory microenvironment [14-16]. Mitochondrial dysfunction is a widely recognized fundamental driver of cellular senescence [17]. Specifically, the disruption of mitochondrial dynamics—tipping the balance towards excessive fission—leads to the accumulation of damaged mitochondria, reactive oxygen species (ROS) production, and cytosolic release of mitochondrial DNA (mtDNA), thereby triggering innate immune responses [18]. In aged or stressed microglia, impaired mitophagy fails to effectively clear these defective organelles [19, 20]. Consequently, the accumulated cytosolic mtDNA robustly activates the cyclic GMP-AMP synthase (cGAS)-stimulator of interferon genes (STING) pathway, which has recently been identified as a critical driver of microglial dysfunction and neuroinflammation in SAH [21, 22]. Dynamin-related protein 1 (Drp1) is the master regulator of mitochondrial fission; its hyperactivity has been implicated in various neurodegenerative diseases [23, 24]. However, whether Drp1-mediated mitochondrial fragmentation drives microglial senescence-like transition in the context of SAH remains to be elucidated.

Immunometabolism has emerged as a critical frontier in understanding the regulation of innate immunity [25, 26]. Immune-responsive gene 1 (IRG1), also known as ACOD1, is the enzyme responsible for the production of itaconate from the TCA cycle intermediate cis-aconitate [27]. Itaconate acts as a vital immunometabolic brake, exerting anti-inflammatory and antioxidant effects [28]. It functions primarily by modifying cysteine residues on target proteins *via* alkylation, thereby altering protein structure and function [29]. Specifically, itaconate has been shown to alkylate KEAP1, thereby activating the transcription factor Nrf2 to induce antioxidant gene expression [30]. Furthermore, itaconate modifies cysteine 548 on NLRP3, which disrupts the NLRP3-NEK7 interaction and consequently inhibits inflammasome activation [31]. Recent studies have identified itaconate as a distinct aging-associated metabolite, with its accumulation in peripheral tissues reflecting age-related immune alterations, and studies have demonstrated its therapeutic efficacy in mitigating neuroinflammation in age-related disorders such as postoperative cognitive dysfunction [32, 33]. However, the specific role and underlying mechanism of the IRG1/itaconate axis in regulating the senescence-like transition of microglia following SAH remain to be elucidated.

In the present study, we investigated the role of the IRG1/itaconate axis in modulating microglial phenotypes following SAH. We identified that the upregulation of IRG1 and itaconate is a protective endogenous response to SAH that is significantly blunted in aged mice. Mechanistically, we demonstrate that itaconate exerts its neuroprotective effects by directly alkylating the small GTPase RhoA at the cysteine 107 (C107) residue. This modification inhibits the activation of the RhoA/ROCK1 signaling pathway, subsequently suppressing Drp1-dependent mitochondrial fission. Consequently, this cascade prevents the senescence-like transition of microglia and attenuates neuroinflammation. Our findings unveil a novel metabolic-mitochondrial checkpoint involving the IRG1/itaconate-RhoA-Drp1 axis, providing a potential therapeutic strategy to combat age-related exacerbation of brain injury following SAH.

## MATERIALS AND METHODS

### Animals

This study primarily employed male C57BL/6 mice at the age of either 10 weeks or 10 months, while 10-month-old female mice were also included for key validation experiments to address potential sex differences. The 10-month-old group was specifically selected to model middle age, a critical transitional phase marking the onset of microglial priming and early immunosenescence [34]. This age point enables the investigation of age-related immune shifts following SAH while mitigating the confounding effects of extreme frailty and excessively high surgical mortality typically associated with advanced senescence (> 18 months). Microglia-specific IRG1 knockout (cKO) mice were generated through the crossbreeding of Cx3cr1-Cre tool mice with IRG1^flox/flox^ mice. All animal experiments were executed in compliance with the Guide for the Care and Use of Laboratory Animals issued by the National Institutes of Health, and prior approval was obtained from the Animal Welfare and Ethical Committee of Shanghai Rat & Mouse Biotech Co., Ltd. (Approval Number: 2022093023). The mice were housed in well-ventilated cages within a pathogen-free facility, where the environmental conditions were maintained at a constant temperature of 23 ± 1°C and a relative humidity ranging from 45% to 55%, along with a 12-hour light-dark circadian cycle. Both food and water were provided ad libitum.

### SAH model

The murine SAH model was established utilizing the endovascular perforation technique. Animals were anesthetized via isoflurane inhalation. A midline cervical incision was performed to expose the left carotid arterial system. The left common carotid artery and left external carotid artery were then ligated. A nylon monofilament was inserted 1 cm distal to the bifurcation of the external carotid artery. The filament was advanced approximately 2 mm upon encountering resistance, maintained in position for 10 seconds post-perforation, and subsequently withdrawn at a constant rate. The sham-operated group underwent an identical surgical protocol with the exception of the endovascular perforation step. The severity of SAH and hematoma volume were assessed using validated SAH grading scales [35]. To ensure model consistency and sufficient injury severity, animals exhibiting an SAH grade of 7 or lower were excluded from further analyses.

### *In vivo* administration in mice

4-octyl itaconate (4-OI), mitochondrial division inhibitor 1 (Mdivi-1), and GSK429286A were each formulated as sterile injectable solutions using a mixed solvent composed of 10% dimethyl sulfoxide (DMSO) and 20% sulfobutyl ether-β-cyclodextrin (SBECD). Thirty minutes after the establishment of the SAH model, the mice received intraperitoneal injections of 4-OI at a dose of 100 mg/kg, Mdivi-1 at 25 mg/kg, and GSK429286A at 25 mg/kg, respectively. Mice in the control group were injected with an equal volume of the vehicle solution to serve as a baseline for statistical comparisons and to rule out any potential physiological effects introduced by the mixed solvent.

### Short-term neurological function assessment

To assess acute neurological impairments following SAH, three standardized behavioral tests—namely the modified Garcia score, beam balance score, and grip test score—were administered 24 hours post-SAH induction [36, 37]. The modified Garcia score quantified neurological function through six parameters: spontaneous locomotor activity, symmetry of limb movement, forelimb extension capacity, climbing proficiency, trunk proprioception, and vibrissae-mediated reflex responses, with higher scores indicating better neurological function. For the beam balance score, mice were positioned on a horizontal wooden beam, and their motor performance was evaluated based on their ability to maintain balance and traverse the beam over a 60-second period. In the grip test score, mice were placed on a horizontal wooden pole, and performance was graded based on both the number of limbs engaged and the strength of their grip on the pole surface.

### Brain water content detection

To evaluate cerebral edema and tissue injury subsequent to SAH, brain water content was quantified. At 24 hours post-SAH, murine brains were excised and separated into ipsilateral and contralateral hemispheres. The wet weight of the ipsilateral hemisphere was immediately measured, after which the tissue was dried in an oven at 105 °C for 24 hours to determine the dry weight. Brain water content was computed as a percentage using the following equation: [(wet weight - dry weight)/wet weight] × 100% [35, 38].

### Nissl staining

Nissl staining was employed to evaluate neuronal survival and mortality. Specifically, 20 μm-thick coronal frozen sections were thoroughly rehydrated with PBS and then immersed in 0.1% toluidine blue staining solution (Solarbio, Beijing, China), followed by incubation in a constant-temperature water bath at 55 °C for 15 minutes. This staining solution was freshly prepared with 0.1 M acetate buffer (pH 4.5) to maintain optimal staining efficiency. After staining, the sections were quickly rinsed with double-distilled water and immediately transferred to 70% ethanol for gradient differentiation to eliminate background staining. Visualization and image capture were performed using a light microscope (Olympus Optical, Japan). The quantification of viable neurons within the ipsilateral temporal cortex was conducted via ImageJ software.

### Fluoro-Jade C staining

To identify degenerating or dead neurons subsequent to SAH, Fluoro-Jade C (FJC) staining was performed using an adapted protocol from the FJC Ready-to-Dilute Staining Kit (Biosensis, United States). Fluorescent micrographs were acquired with a fluorescence microscope (Olympus Optical, Japan), facilitating the clear visualization of FJC-labeled neurons. Quantitative analysis of FJC-positive neuronal proportions within the ipsilateral temporal cortex was subsequently conducted using ImageJ software, which served as a metric for evaluating neuronal degeneration or death post-SAH.

### Long-term neurological function assessment

To assess long-term spatial learning and memory capabilities, the Morris water maze test was implemented from post-SAH days 22 to 26, consistent with established protocols in prior studies [35]. Mice underwent a 5-second platform habituation period on days 22 through 25 as part of the cued training phase. Subsequent spatial acquisition trials involved releasing the mice from multiple semi-randomized starting positions and requiring them to locate a submerged platform within a 60-second timeframe. Locomotor activity and swimming patterns were recorded via a video monitoring system, with parameters such as swim distance, duration, and path trajectory analyzed using a computerized tracking system. On day 25, escape latency—defined as the elapsed time from trial initiation to platform arrival—was measured. A probe trial was conducted on day 26, during which the platform was removed, enabling free exploration of the former platform location. The percentage of total swimming distance and time spent within the target quadrant were quantified. Escape latency, target quadrant distance percentage, and target quadrant time percentage were employed as primary outcome measures to evaluate spatial learning and memory performance in the murine model.

### Cell culture

BV2 microglial cells were procured from Yuchun Biology (Shanghai, China). The cells were maintained in high-glucose Dulbecco's Modified Eagle Medium (DMEM) (Cytiva, USA), which was supplemented with 10% fetal bovine serum (Cellmax, Beijing, China) and 1% penicillin-streptomycin solution (Solarbio, Beijing, China). All cell cultures were incubated at 37 °C within a humidified atmosphere containing 5% carbon dioxide. Oxyhemoglobin (OxyHb) was prepared from hemoglobin (Sigma-Aldrich, USA) following the manufacturer's recommended protocol. To establish an in vitro model of SAH, the culture medium was supplemented with OxyHb at a final concentration of 20 μM, and the cells were exposed to this condition for 24 hours. In the experimental groups, cells were treated with either 250 μM 4-OI or 10 μM GSK429286A, with a DMSO vehicle control group included for comparison.

### IRG1 knockdown *in vitro*

For in vitro knockdown of IRG1, BV2 cells were transfected with a lentiviral vector encoding short hairpin RNA targeting IRG1 (shIRG1; Vigene Biology, Shandong, China). At 72 hours post-infection, transfected cells were selected using 3 µg/mL puromycin and then subcultured for subsequent experimental procedures. The efficiency of IRG1 silencing was verified through quantitative real-time polymerase chain reaction (qPCR) analysis. The nucleotide sequences of the shIRG1 constructs were as follows: forward: 5′-CCGGCCTGA CGTCCAGTACGTAAACCTCGAGGTTTACGTACGGACGTCAGGTTTTTG-3′ and reverse: 3′-GGCCGG ACTGCAGGTCATGCATTTGGAGCTCCAAATGCATGACCTGCAGTCCAAAAAC-5′.

### Mitochondrial morphological analysis

Mitochondrial fission was evaluated using the Mito-Tracker Red CMXRos fluorescent probe (Beyotime, Shanghai, China). Cells were incubated with 100 nM Mito-Tracker Red CMXRos in PBS at 37°C for 30 minutes. Subsequent visualization was performed using a fluorescence microscope (Olympus Optical, Japan), and mitochondrial structural parameters, including average branch length and median branch length, were quantified using ImageJ software.

### Mitochondrial membrane potential assessment

Mitochondrial membrane potential was determined utilizing the JC-1 assay kit (Beyotime, Shanghai, China). Cells were exposed to JC-1 dye (10 μg/mL) in serum-free medium for 20 minutes at 37°C. Following thorough washing, fluorescent signals were detected via microscopy (Olympus Optical, Japan). A shift toward increased green fluorescence intensity accompanied by a reduction in red fluorescence intensity was interpreted as a decline in mitochondrial membrane potential. Fluorescence quantification was performed using ImageJ software.

### Mitochondrial membrane permeability measurement

Mitochondrial membrane permeability was assessed by quantifying the release of mtDNA into the cytoplasm via qPCR. Mitochondrial and cytosolic fractions were isolated using the Cell Mitochondria Isolation Kit (Beyotime, Shanghai, China). Genomic DNA was extracted from the cytosolic fraction using the TIANamp Genomic DNA Kit (TIANGEN, Beijing, China). The relative abundance of mtDNA was measured using primers specific to the mitochondrial D-loop region, with normalization against nuclear DNA encoding telomerase reverse transcriptase (TERT) (Supplementary Table 1).

### Liquid chromatography-tandem mass spectrometry

Liquid chromatography-tandem mass spectrometry (LC-MS/MS) quantified metabolic changes in post-SAH mouse brain tissues. Ipsilateral temporal cortex was flash-frozen in liquid nitrogen, homogenized in pre-cooled methanol-water (4:1, v/v, 0.1% formic acid) at 30 Hz for 2 min (ice-bath cooled), centrifuged (15,000×g, 4°C, 20 min), filtered (0.22 μm), and spiked with isotope-labeled internal standards (low-temperature processing maintained). Separation used Waters ACQUITY UPLC HSS T3 column (2.1×100 mm, 1.8 μm). MS employed negative-ion electrospray ionization with optimized parameters; multiple reaction monitoring with validated collision energies and internal standard calibration enabled quantification. Isotope dilution standard curves with linearity (r² ≥ predefined criteria) and MRM transitions ensured accurate quantification/qualification. A quality control sample was created by pooling equal volumes from each sample to evaluate the reproducibility of the LC-MS/MS analysis.

### qPCR

Total RNA was extracted from the specimens using TRIzol reagent (Invitrogen, USA) in strict accordance with the manufacturer's instructions. Subsequently, complementary DNA (cDNA) synthesis was performed in 0.2 mL PCR tubes using the PrimeScript RT Master Mix (Takara). The reaction mixture consisted of 1 μg of total RNA, 4 μL of 5× RT Master Mix, and RNase-free water to bring the final volume to 20 μL. Reverse transcription was carried out by incubating the mixture at 42 °C for 30 minutes, followed by heating at 85 °C for 5 seconds to inactivate the reverse transcriptase enzyme. The synthesized cDNA was then analyzed via quantitative polymerase chain reaction with SYBR Green PCR Master Mix (Toyobo, Japan) on a Bio-Rad CFX96 real-time PCR system, adhering to the prescribed protocol. Gene expression levels were determined using the 2^-ΔΔCt^ method, with relative mRNA abundances normalized to the housekeeping gene β-actin. The specific primers employed for qPCR are detailed in Supplementary Table 1.

### Western blot

For total protein analysis, brain tissue or cellular samples were lysed with RIPA buffer (Beyotime, China). To specifically assess Drp1 mitochondrial translocation, mitochondrial fractions were independently isolated using a mitochondrial extraction kit (Beyotime, China) prior to lysis. Homogenates were centrifuged at 12,000 rpm for 20 min at 4°C, and supernatants were collected. Protein concentration was quantified using the BCA assay kit (Thermo Scientific, USA). Denatured proteins (30 μg/sample) were separated by SDS-PAGE on precast gels (Epizyme Biotech, China) at 120 V constant voltage, then electrotransferred onto PVDF membranes (Millipore, USA). Membranes were blocked with TBST containing 5% non-fat milk for 1 h at room temperature, followed by overnight incubation at 4°C with primary antibodies diluted in blocking solution (Supplementary Table 2). β-actin and COX IV were used as loading controls for total protein and mitochondrial fractions, respectively. After three TBST washes (10 mM Tris, 150 mM NaCl, 0.05% Tween-20, pH 7.5), membranes were incubated with HRP-conjugated secondary antibodies (goat anti-mouse/rabbit IgG, Cat. No. bs-0296Gs/bs-0295Gs, 1:5000, Bioss, China) for 1 h at room temperature. Protein bands were visualized using ECL substrate (Thermo Scientific) and imaged with the Odyssey gel imaging system (LI-COR).

### Immunofluorescence staining

Coronal cryosectioned brain tissue sections were rinsed in PBS for 10 minutes, followed by 20-minute permeabilization with PBS containing 0.5% Triton X-100 (Sigma-Aldrich, USA). To reduce non-specific binding, sections were blocked with 5% bovine serum albumin (Sigma-Aldrich, USA) for 1 hour at room temperature. Specific primary antibodies, diluted in blocking buffer (Supplementary Table 2), were applied overnight at 4°C. To validate antibody specificity and exclude non-specific staining, parallel control experiments were performed under identical conditions: (1) isotype antibody controls, where primary antibodies were replaced with Mouse IgG Isotype Control or Rabbit IgG Isotype Control (share-bio, Cat. No. SB-A00001/SB-A00002, Shanghai, China); (2) secondary antibody only controls, where sections were incubated with Alexa Fluor-labeled secondary antibodies (Beyotime, Cat. No. A0428/A0453, Shanghai, China) without primary antibody incubation; (3) tissue minus/plus controls, where skeletal muscle tissue from normal young mice (tissue minus control, lacking the target antigen) and brain tissue from LPS-administered mice (tissue plus control, with expected target antigen expression) were processed alongside experimental samples. After primary incubation, sections were washed three times with PBS at room temperature to remove unbound antibody, then incubated with corresponding Alexa Fluor-labeled secondary antibodies (Beyotime, Cat. No. A0428/A0453, Shanghai, China) for 1.5 hours. Post-secondary incubation, sections were washed again with PBS, mounted on glass slides, and coverslipped using antifade mounting medium containing 4',6-diamidino-2-phenylindole (DAPI) for nuclear counterstaining. Immunofluorescent sections were visualized with a fluorescence or laser scanning confocal microscope (Olympus Optical, Japan), and images were quantitatively analyzed using ImageJ software.

### Detection of itaconate-protein interaction

To investigate the interactions between itaconate and target proteins, we utilized iTalk, a structural analog of itaconate functionalized with a terminal alkyne moiety [29]. Cultured BV2 microglia were incubated with 100 μM iTalk for 4 hours, allowing the probe to effectively penetrate the cells and bind to potential target proteins. Following incubation, total proteins were extracted using RIPA lysis buffer supplemented with 1% Triton X-100 and protease inhibitors to preserve protein integrity. Biotin-azide was then covalently conjugated to the alkyne group of the probe via a click chemistry reaction. Subsequently, the biotinylated target proteins were enriched from the cell lysates using streptavidin-conjugated magnetic beads. Finally, the enriched proteins were analyzed by Western blotting to verify the specific interaction between the candidate protein and itaconate.

### Mass spectrometric identification of RhoA cysteine alkylation

To characterize the alkylation of RhoA cysteine residues, 293T cells overexpressing RhoA were treated with 1 mM itaconate or 250 μM 4-OI for 6 hours to optimize modification efficiency and cell viability. Following lysis, 50 μg of protein was resolved via SDS-PAGE using 5% stacking and 12% resolving gels. Electrophoresis was conducted at 80 V, increasing to 120 V in approximately 90 minutes. Gels were stained with 0.1% Coomassie Brilliant Blue R-250 and destained in methanol/acetic acid. Guided by the 21 kDa marker, the 18-25 kDa region was excised to capture RhoA and potential post-translational migration shifts. Bands were preserved in 50% methanol containing 1% acetic acid and analyzed by mass spectrometry. Cysteine residues were screened for mass shifts corresponding to itaconate (+130.10 Da, C5H6O4) or 4-OI (+242.1518 Da, C13H22O4) to pinpoint modification sites.

### Site mutation

To validate the functional relevance of identified alkylation sites, site-directed mutagenesis was performed to generate RhoA mutants by substituting cysteines at positions 16, 107, and 159 with serine. Using the pCMV3-RhoA vector, overlap extension PCR was conducted with high-fidelity polymerase, followed by DpnI digestion of the parental template and transformation into E. coli DH5α. Sanger sequencing of independent clones confirmed that precise TGT/TGC to TCT substitutions were achieved without introducing random mutations. The resulting plasmids were designated pCMV3-RhoA-C16S, pCMV3-RhoA-C107S, and pCMV3-RhoA-C159S. Subsequently, to evaluate protein stability, BV2 microglial cells were transfected with wild-type or mutant plasmids using a liposomal method. Western blot analysis performed 48 hours post-transfection demonstrated that all mutants exhibited molecular weights and expression levels comparable to wild-type RhoA, confirming that these point mutations did not compromise protein stability.

### Statistical analysis

The data are expressed as mean ± SEM. Prior to hypothesis testing, the Shapiro-Wilk test was performed to assess data normality using SPSS 24.0 software. For two-group comparisons: unpaired Student’s t-test was applied if data conformed to a normal distribution; the Mann-Whitney U test (non-parametric alternative) was used when the normality assumption was violated. For comparisons involving three or more groups: one-way analysis of variance (ANOVA) was employed for normally distributed data, while the Kruskal-Wallis H test (non-parametric alternative) was adopted for non-normally distributed data. All statistical calculations were executed using SPSS 24.0 software. GraphPad Prism 8.0 was utilized to generate histograms for data visualization. A P-value < 0.05 was considered statistically significant. Significance levels are denoted as follows: *, P < 0.05; **, P < 0.01; ***, P < 0.001; ****, P < 0.0001; ns, not significant. Exact P-values for all statistical comparisons, along with the specific test applied in each case, are summarized in Supplementary Table 3.

## RESULTS

### The IRG1/itaconate axis is activated following SAH but is impaired by aging

We initially characterized the spatiotemporal expression profiles of IRG1 and itaconate in brain tissues following SAH. Western blot and qPCR analyses revealed that IRG1 expression levels in the ipsilateral temporal lobe were significantly upregulated after SAH, peaking at 6 hours post-injury (Fig. 1A-B). Consistent with this, LC-MS/MS analysis indicated that itaconate levels followed a similar temporal trend to IRG1, reaching a peak between 6 and 12 hours (Fig. 1C). We next examined the spatial distribution of these changes at 6 hours post-SAH. The results demonstrated that both IRG1 and itaconate were upregulated in the cerebral hemispheres and cerebellum, with the most pronounced elevation observed in the ipsilateral hemisphere; however, no significant upregulation was detected in the brainstem (Fig. 1D-H). Immunofluorescence staining further localized the upregulated IRG1 primarily to microglia. Consistent with the western blot and qPCR data, microglial IRG1 expression was significantly increased following SAH (Fig. 1I-J).

**Figure 1.**
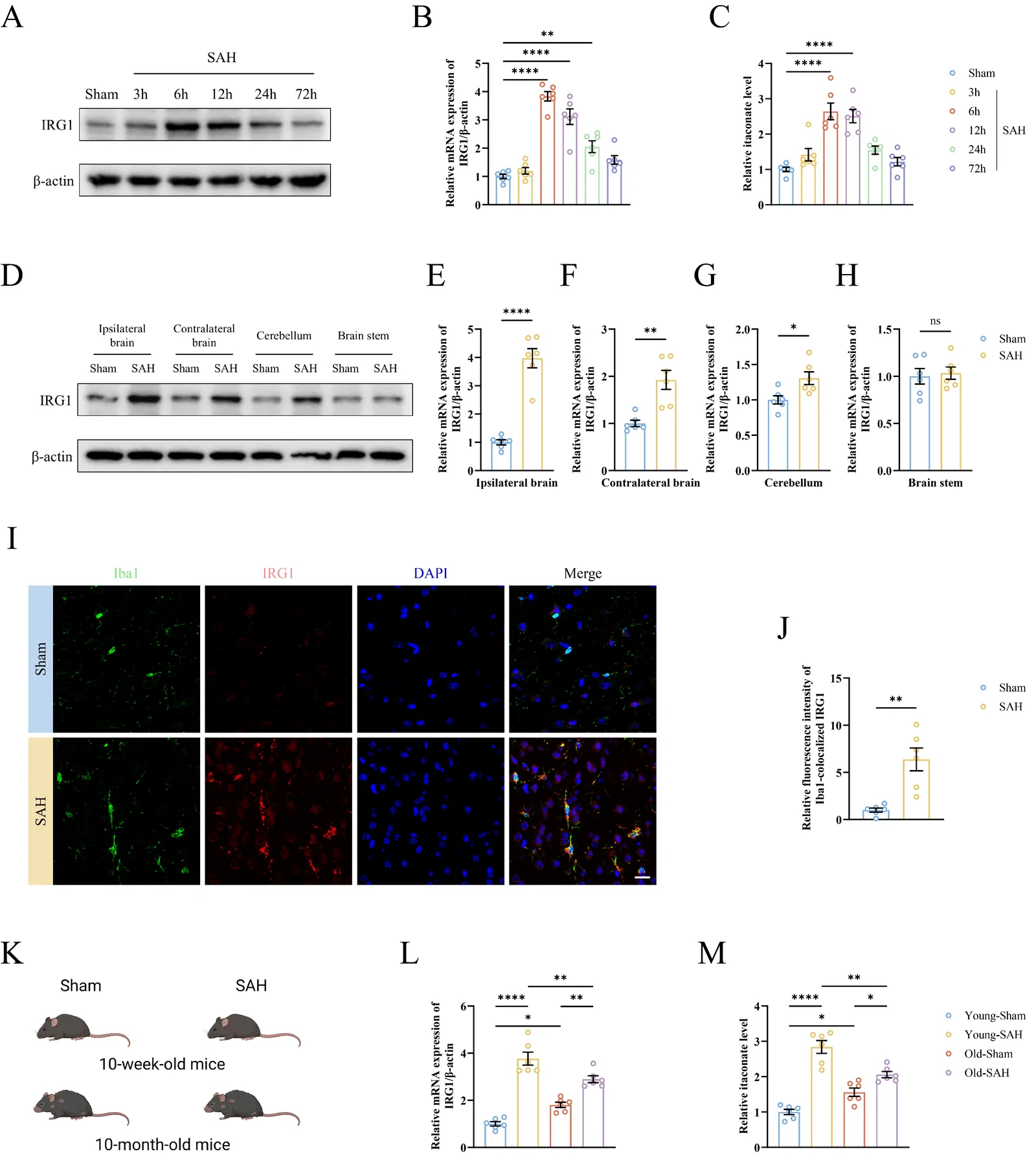
**IRG1 and itaconate expressions are increased with age-negative correlation after SAH**. (**A-B**) Western blot and qPCR (n=6 mice per group) to evaluate IRG1 expression changes in the ipsilateral temporal lobe at various time points following SAH. (**C**) LC-MS/MS to evaluate itaconate level changes in the ipsilateral temporal lobe at various time points following SAH (n=6 mice per group). (**D-H**) Western blot and qPCR (n=6 mice per group) to evaluate IRG1 expression changes in different brain regions at 6 hours after SAH. (**I-J**) Immunofluorescence staining to evaluate IRG1 expression and its co-localization with microglia in the ipsilateral temporal lobe after SAH (scale bar=20μm; n=6 mice per group). (**K-M**) qPCR and LC-MS/MS to compare the changes in IRG1 expression and itaconate level after SAH between young and aged mice (n=6 mice per group). Band intensities were quantified using ImageJ and normalized to the loading control. Relative mRNA levels were calculated using the 2^-ΔΔCt^ method. Image analysis was performed in a blinded manner. Data are presented as mean ± SEM, normalized to the first group, and analyzed by Student's t-test (E-H, J) or One-way ANOVA (B-C, L-M).

**Figure 2.**
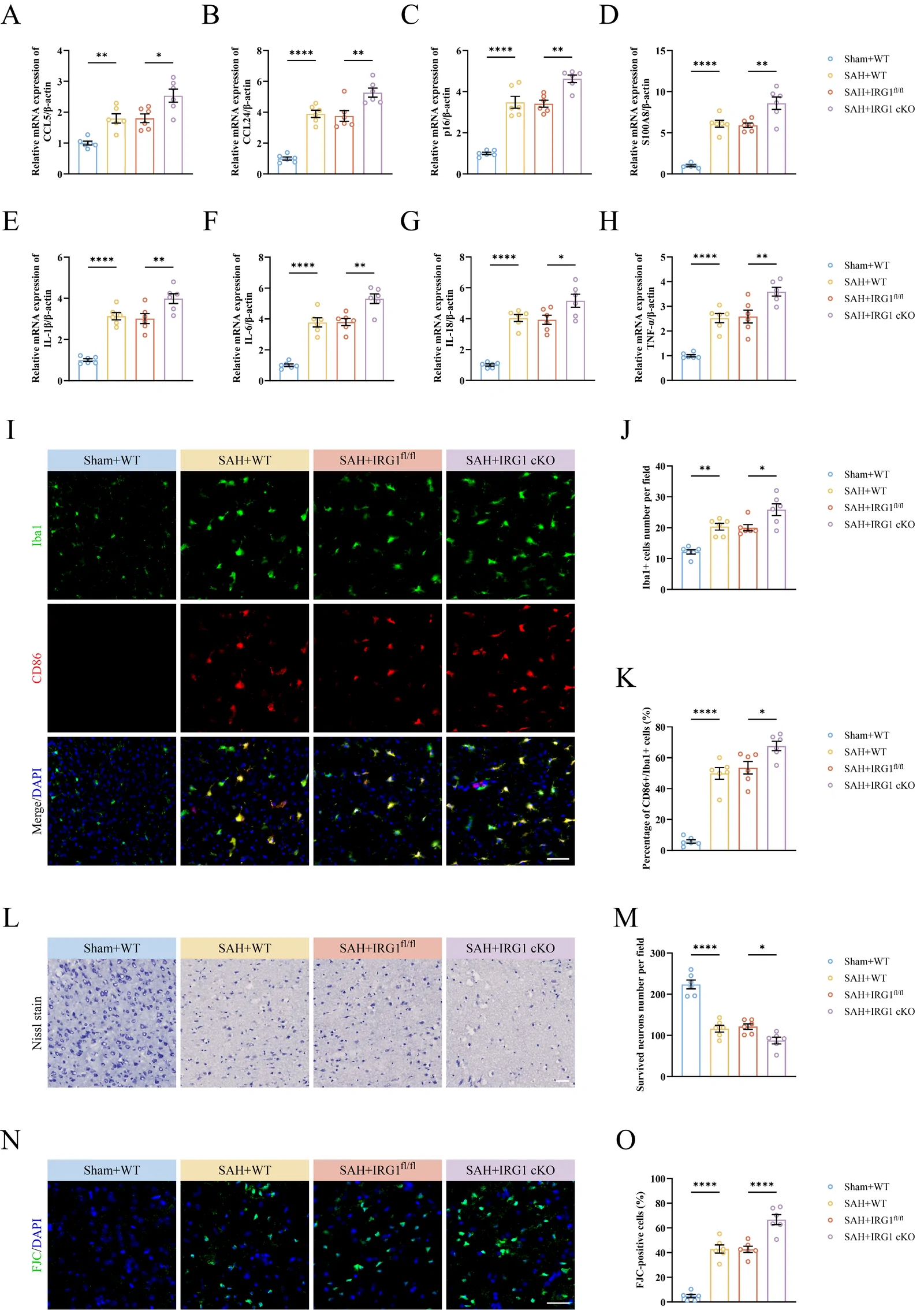
**IRG1 deficiency exacerbates microglial senescence-like transition and brain injury after SAH**. (**A-H**) qPCR to evaluate CCL5, CCL24, p16, S100A8, IL-1β, IL-6, IL-18, and TNF-α expressions in mice of Sham+WT, SAH+WT, SAH+IRG1^fl/fl^, and SAH+IRG1 cKO groups (n=6 mice per group). (**I-K**) Immunofluorescence staining to evaluate microglial number and polarization (scale bar=60μm; n=6 mice per group). (**L-M**) Nissl staining to assess neuronal survival (scale bar=40μm; n=6 mice per group). (**N-O**) FJC staining to assess neuronal degeneration and death (scale bar=40μm; n=6 mice per group). Relative mRNA levels were calculated using the 2^-ΔΔCt^ method. Image analysis was performed in a blinded manner. Data are presented as mean ± SEM, normalized to the first group, and analyzed by One-way ANOVA (A-H, J-K, M, O).

To investigate the age-dependence of this response, we compared IRG1 and itaconate dynamics in young (10-week-old) and aged (10-month-old) mice (Fig. 1K). Under physiological conditions, aged mice exhibited higher basal levels of IRG1 and itaconate compared to young mice. However, upon SAH induction, although IRG1 and itaconate levels increased in aged mice, the magnitude of this upregulation was significantly blunted compared to their young counterparts (Fig. 1L-M). These findings suggest that aging impairs the robust activation of the IRG1/itaconate axis following SAH, which may contribute to the exacerbated inflammatory response observed in the aged brain.

### The IRG1/itaconate axis inhibits microglial senescence-like transition and attenuates brain injury after SAH

Microglial senescence-like transition is a critical driver of neuroinflammation and brain injury [14-16]. To explore the role of the IRG1/itaconate axis in this process, we utilized 10-month-old mice for subsequent experiments. We first generated microglia-specific IRG1 knockout mice. Importantly, qPCR analysis revealed that IRG1 deficiency did not significantly alter the baseline expression of senescence-associated markers or pro-inflammatory cytokines under physiological conditions (Supplementary Fig. 1A-H), ruling out pre-existing developmental defects. In contrast, under SAH conditions, IRG1 deficiency further exacerbated the upregulation of senescence-associated markers, including CCL5, CCL24, p16, and S100A8, indicating an accelerated senescence-like transition (Fig. 2A-D). Furthermore, IRG1 deletion potentiated the expression of pro-inflammatory cytokines (IL-1β, IL-6, IL-18, and TNF-α), increased the number of Iba1-positive microglia, and elevated the proportion of CD86-positive microglia, suggesting aggravated neuroinflammation (Fig. 2E-K). Complementing these findings, qPCR analysis confirmed that IRG1 deficiency promoted the expression of pro-inflammatory markers CD86 and iNOS. Notably, while IRG1 deletion did not alter the expression of the anti-inflammatory marker CD206, it significantly suppressed the levels of the anti-inflammatory cytokine IL-10 (Supplementary Fig. 2A-D).

We next assessed the impact of microglial IRG1 deficiency on neurological function and brain injury. At 24 hours post-SAH, IRG1 cKO mice exhibited significantly lower modified Garcia scores, beam balance scores, and grip test scores compared to control littermates (Supplementary Fig. 2E-G), accompanied by increased brain water content (Supplementary Fig. 2H). Nissl and FJC staining revealed that IRG1 deficiency significantly exacerbated neuronal loss and increased the proportion of degenerating neurons (Fig. 2L-O). In the Morris water maze test, IRG1 cKO mice displayed marked deficits in spatial learning and memory, evidenced by prolonged escape latencies during the acquisition phase and reduced time and distance in the target quadrant during the probe trial (Supplementary Fig. 2I-L). These results indicate that microglial IRG1 deficiency exacerbates SAH-induced brain injury and functional deficits. Importantly, treatment with the cell-permeable itaconate derivative 4-OI largely ameliorated the adverse effects of IRG1 deficiency. These included the senescence-like transition of microglia (Supplementary Fig. 3A-D), their shift toward a pro-inflammatory phenotype as well as the suppression of their anti-inflammatory capacity (Supplementary Fig. 3I-K; Supplementary Fig. 4A-D), along with the exacerbation of neuroinflammation (Supplementary Fig. 3E-H), brain injury (Supplementary Fig. 3L-O), and neurological dysfunction (Supplementary Fig. 4E-L). Collectively, these data demonstrate that the IRG1/itaconate axis exerts a protective effect by inhibiting microglial senescence and inflammation, thereby attenuating brain injury.

Given that sex is a critical biological variable that can influence microglial function and the pathophysiological progression of SAH, we further validated our findings in female mice to ensure the generalizability of the IRG1/itaconate axis's protective role. Consistent with the observations in male mice, qPCR analysis revealed that IRG1 deficiency in females significantly exacerbated the SAH-induced upregulation of senescence-associated markers (CCL5, CCL24, p16, and S100A8) and pro-inflammatory cytokines (IL-1β, IL-6, IL-18, and TNF-α) (Supplementary Fig. 5A-H). In terms of neurological outcomes, female IRG1 cKO mice displayed significantly lower modified Garcia scores and beam balance scores at 24 hours post-SAH compared to their control littermates (Supplementary Fig. 5I-J). Although the reduction in grip test scores did not reach statistical significance in the female cohort (Supplementary Fig. 5K), IRG1 deficiency markedly increased brain water content, indicating aggravated cerebral edema (Supplementary Fig. 5L). Collectively, these supplementary data demonstrate that the protective effects of the microglial IRG1/itaconate axis against SAH-induced senescence and neuroinflammation are largely conserved across both sexes, further strengthening the conclusions of our study.

**Figure 3.**
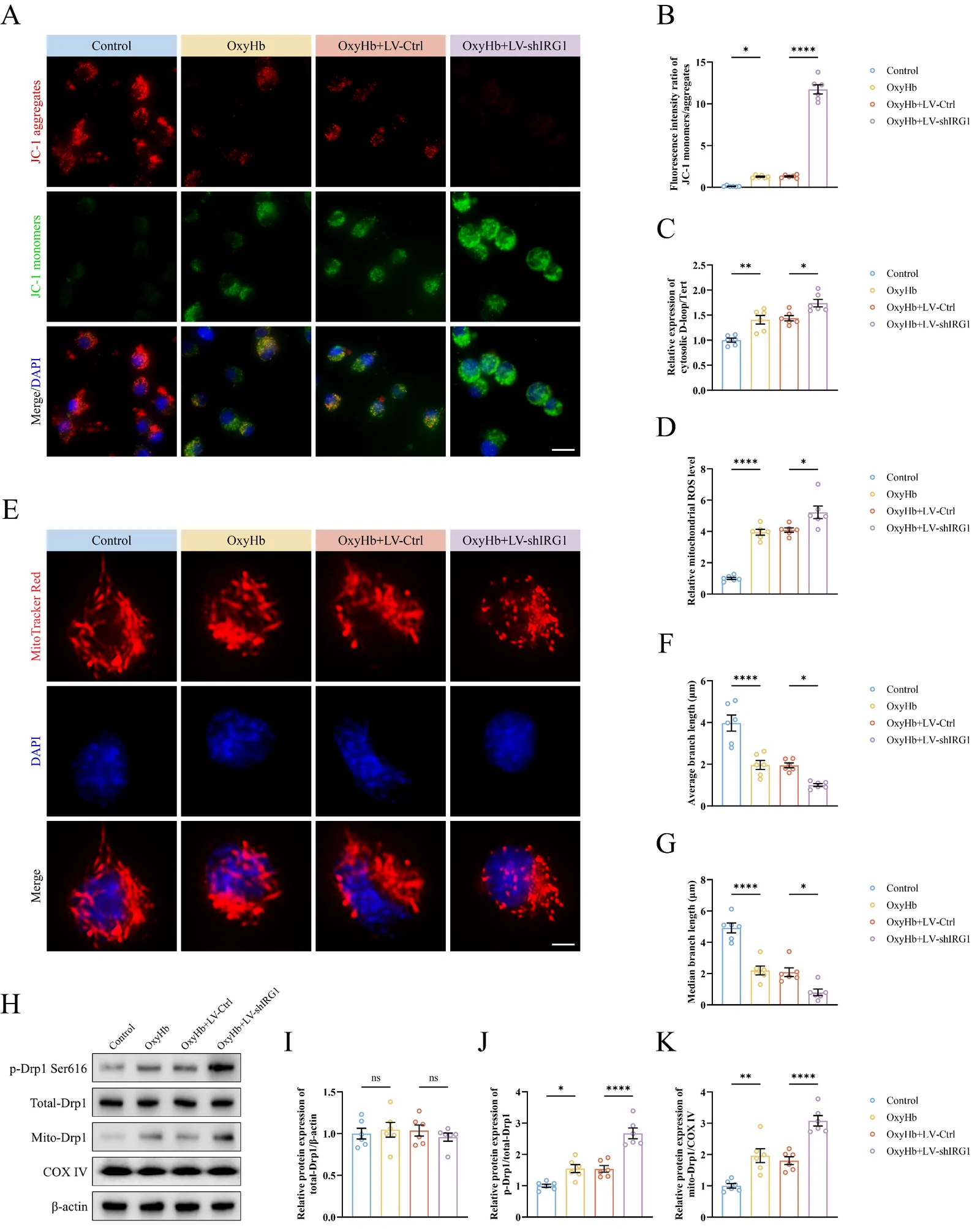
**IRG1 deficiency promotes Drp1-dependent mitochondrial fission and exacerbates mitochondrial dysfunction in OxyHb-stimulated microglia**. (**A-B**) JC-1 fluorescent probe to evaluate mitochondrial membrane potential level in BV2 cells treated with Control, OxyHb, OxyHb+LV-Ctrl, and OxyHb+LV-shIRG1 (scale bar=20μm; n=6 independent biological replicates per group). Fluorescence ratios (Green/Red) were calculated to determine membrane potential. (**C**) qPCR to evaluate cytoplasmic D-loop level (n=6 independent biological replicates per group). (**D**) MitoSOX Red to evaluate mitochondrial ROS level (n=6 independent biological replicates per group). (**E-G**) Mito-Tracker Red CMXRos fluorescent probe to evaluate mitochondrial mean and median branch lengths (scale bar=5μm; n=6 independent biological replicates per group). ﻿Mitochondrial morphology was analyzed using the ImageJ mitochondrial morphology macro. (**H-K**) Western blot to evaluate the phosphorylation activation and mitochondrial translocation of Drp1 (n=6 independent biological replicates per group). Band intensities were quantified using ImageJ and normalized to the loading control. Image analysis was performed in a blinded manner. Data are presented as mean ± SEM, normalized to the first group, and analyzed by One-way ANOVA (B-D, F-G, I-K).

**Figure 4.**
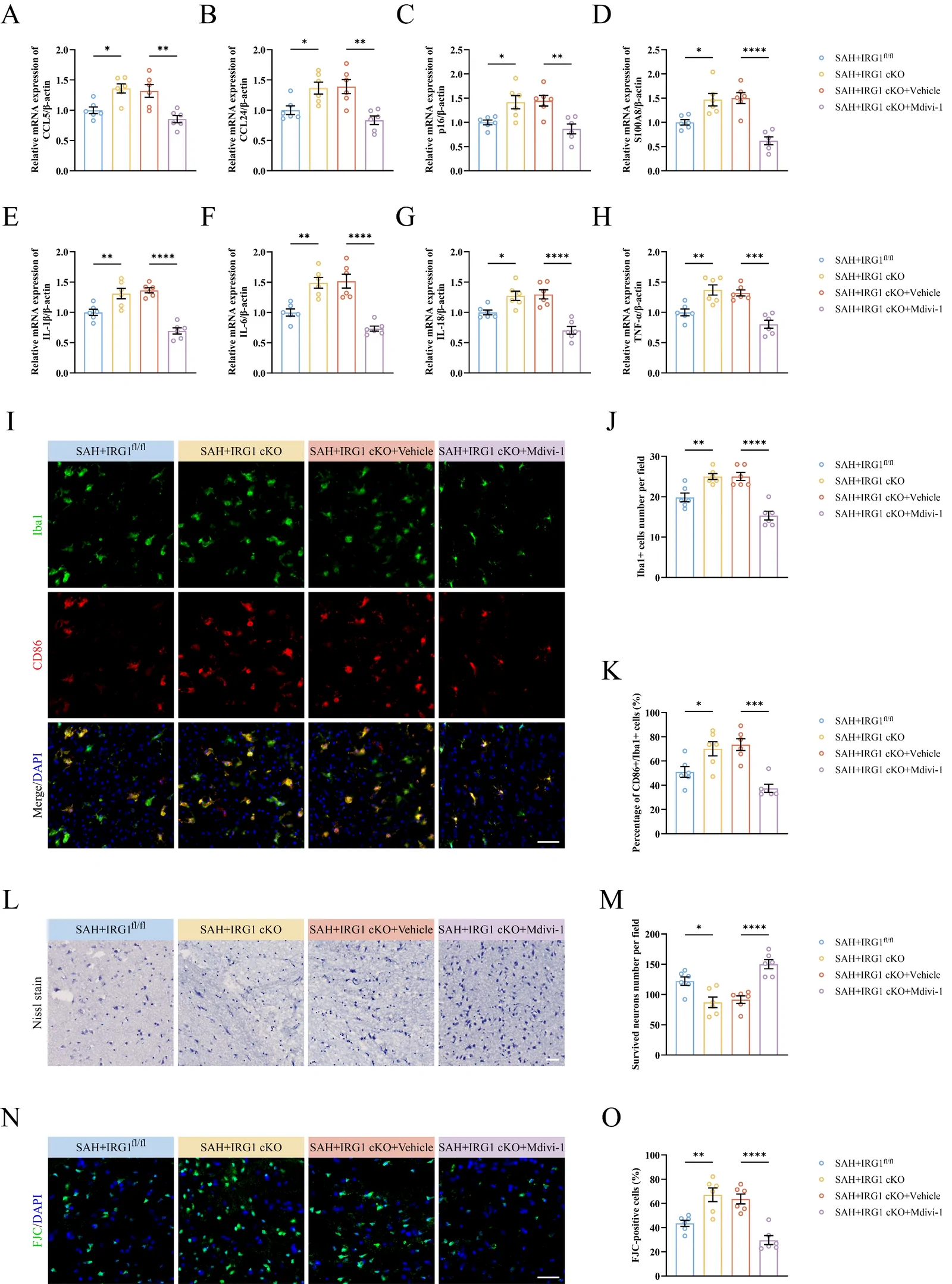
**Mdivi-1 rescues the exacerbation of microglial senescence-like transition and brain injury caused by IRG1 deficiency**. (**A-H**) qPCR to evaluate CCL5, CCL24, p16, S100A8, IL-1β, IL-6, IL-18, and TNF-α expressions in mice of SAH+IRG1^fl/fl^, SAH+IRG1 cKO, SAH+IRG1 cKO+Vehicle, and SAH+IRG1 cKO+Mdivi-1 groups (n=6 mice per group). (**I-K**) Immunofluorescence staining to evaluate microglial number and polarization (scale bar=60μm; n=6 mice per group). (**L-M**) Nissl staining to assess neuronal survival (scale bar=40μm; n=6 mice per group). (**N-O**) FJC staining to assess neuronal degeneration and death (scale bar=40μm; n=6 mice per group). Relative mRNA levels were calculated using the 2^-ΔΔCt^ method. Image analysis was performed in a blinded manner. Data are presented as mean ± SEM, normalized to the first group, and analyzed by One-way ANOVA (A-H, J-K, M, O).

### The IRG1/itaconate axis inhibits Drp1-dependent mitochondrial fission and improves mitochondrial function in microglia

Mitochondrial dysfunction is a hallmark of cellular senescence [17, 18]. To determine whether the IRG1/itaconate axis modulates mitochondrial homeostasis in microglia under SAH conditions, we performed in vitro studies using OxyHb-stimulated BV2 microglia. JC-1 staining revealed that OxyHb stimulation significantly reduced mitochondrial membrane potential, indicating functional impairment. IRG1 knockdown further exacerbated this reduction (Fig. 3A-B). Additionally, qPCR analysis showed that IRG1 deficiency increased the levels of cytosolic mtDNA D-loop, indicative of increased mitochondrial membrane permeability (Fig. 3C). Consistent with these findings, MitoSOX Red fluorescence probe further demonstrated that IRG1 deficiency significantly augmented the elevation of mitochondrial ROS levels triggered by OxyHb stimulation (Fig. 3D).

To characterize mitochondrial morphology, we used Mito-Tracker Red CMXRos staining. IRG1 knockdown aggravated OxyHb-induced mitochondrial fragmentation, as evidenced by reduced mean and median branch lengths (Fig. 3E-G). We then investigated the effect of IRG1 on Drp1, a key regulator of mitochondrial fission [23, 24]. Western blot analysis demonstrated that IRG1 deficiency significantly increased Drp1 phosphorylation at Ser616 and promoted its translocation to mitochondria (Fig. 3H-K). Given that Drp1-mediated excessive fission drives mitochondrial dysfunction in neuroinflammation [39, 40], these results suggest that IRG1 knockdown exacerbates mitochondrial dysfunction by promoting Drp1-dependent fission. Notably, 4-OI treatment reversed the deleterious effects of IRG1 deficiency, including Drp1 activation and translocation (Supplementary Fig. 6A-D), excessive mitochondrial fission (Supplementary Fig. 6E-G), loss of membrane potential (Supplementary Fig. 6H-I), increased membrane permeability (Supplementary Fig. 6J), and elevated mitochondrial ROS levels (Supplementary Fig. 6K). These findings indicate that the IRG1/itaconate axis preserves mitochondrial function by inhibiting Drp1-mediated fission, a mechanism likely central to its anti-senescence and anti-inflammatory properties.

### Inhibition of mitochondrial fission rescues the exacerbation of microglial senescence-like transition and brain injury caused by IRG1 deficiency

To determine whether the protective effects of the IRG1/itaconate axis are mediated through the inhibition of Drp1-dependent fission, we employed the mitochondrial division inhibitor Mdivi-1. Mdivi-1 treatment effectively rescued the upregulation of senescence markers (CCL5, CCL24, p16, and S100A8) induced by IRG1 deficiency following SAH (Fig. 4A-D). Concurrently, it mitigated the aberrant microglial proliferation and pro-inflammatory shift, successfully rescuing the microglial anti-inflammatory function compromised by the loss of IRG1 (Fig. 4E-K; Supplementary Fig. 7A-D).

In addition, Mdivi-1 administration alleviated the aggravated neurological deficits and brain edema observed in microglia-specific IRG1 cKO mice (Supplementary Fig. 7E-H). Histological assessment using Nissl and FJC staining confirmed that Mdivi-1 attenuated the increased neuronal death and degeneration associated with IRG1 deficiency (Fig. 4L-O). These results confirm that IRG1 deficiency exacerbates microglial senescence and neuroinflammation by promoting excessive mitochondrial fission, suggesting that the IRG1/itaconate axis exerts neuroprotection primarily by restraining Drp1-mediated mitochondrial dynamics.

### RhoA/ROCK1 signaling mediates the regulatory role of the IRG1/itaconate axis in Drp1-dependent mitochondrial fission

While our data suggest that the IRG1/itaconate axis inhibits Drp1-dependent fission, the underlying molecular mechanism remained to be elucidated. Itaconate is known to modify protein structure and function via cysteine alkylation [29]. To identify potential targets, we constructed a protein-protein interaction (PPI) network for Drp1, identifying 82 molecules associated with Drp1 signaling (Fig. 5A; Supplementary Supplementary Table 4). Intersection analysis of these molecules with a dataset of 1,926 potential itaconate alkylation targets identified in previous studies (https://pubs.acs.org/ doi/10.1021/jacs.9b11962) (Fig. 5B; Supplementary Supplementary Table 5) yielded 22 candidate proteins (Fig. 5C). Notably, RhoA, ROCK1, ROCK2, and Drp1 were among the candidates. Given the established role of the RhoA/ROCK1 pathway in Drp1 activation [41, 42], we hypothesized that itaconate might indirectly regulate Drp1 via this pathway.

To verify direct interaction, we used an iTalk probe, an alkyne-tagged itaconate analog enabling click chemistry-based enrichment [29]. In OxyHb-stimulated BV2 cells, the iTalk probe significantly enriched RhoA, but not ROCK1, ROCK2, or Drp1, suggesting that itaconate directly binds to RhoA (Fig. 5D). Furthermore, competitive binding assays demonstrated that the addition of exogenous itaconate dose-dependently inhibited the interaction between the iTalk probe and RhoA (Fig. 5E), confirming the specificity of this binding. We then assessed the impact of itaconate on RhoA/ROCK1 signaling using 4-OI. Western blot analysis showed that while OxyHb increased RhoA and ROCK1 expression, as well as RhoA-GTP binding activity and ROCK1 phosphorylation, 4-OI treatment significantly suppressed RhoA-GTP levels and ROCK1 phosphorylation without altering their total protein levels (Fig. 5F-J). To confirm the functional link between this pathway and Drp1, we used the specific ROCK1 inhibitor GSK429286A. Although GSK429286A did not affect total Drp1 levels, it significantly reversed the increased mitochondrial Drp1 accumulation induced by IRG1 knockdown (Fig. 5K-M). Moreover, GSK429286A rescued the exacerbated mitochondrial fission (Fig. 5N-P), loss of membrane potential, increased permeability, and elevated mitochondrial ROS levels (Supplementary Fig. 8A-D) caused by IRG1 deficiency. These data suggest that itaconate alkylates RhoA to inhibit its GTP-binding activity and subsequent ROCK1 phosphorylation, thereby suppressing Drp1-dependent mitochondrial fission.

**Figure 5.**
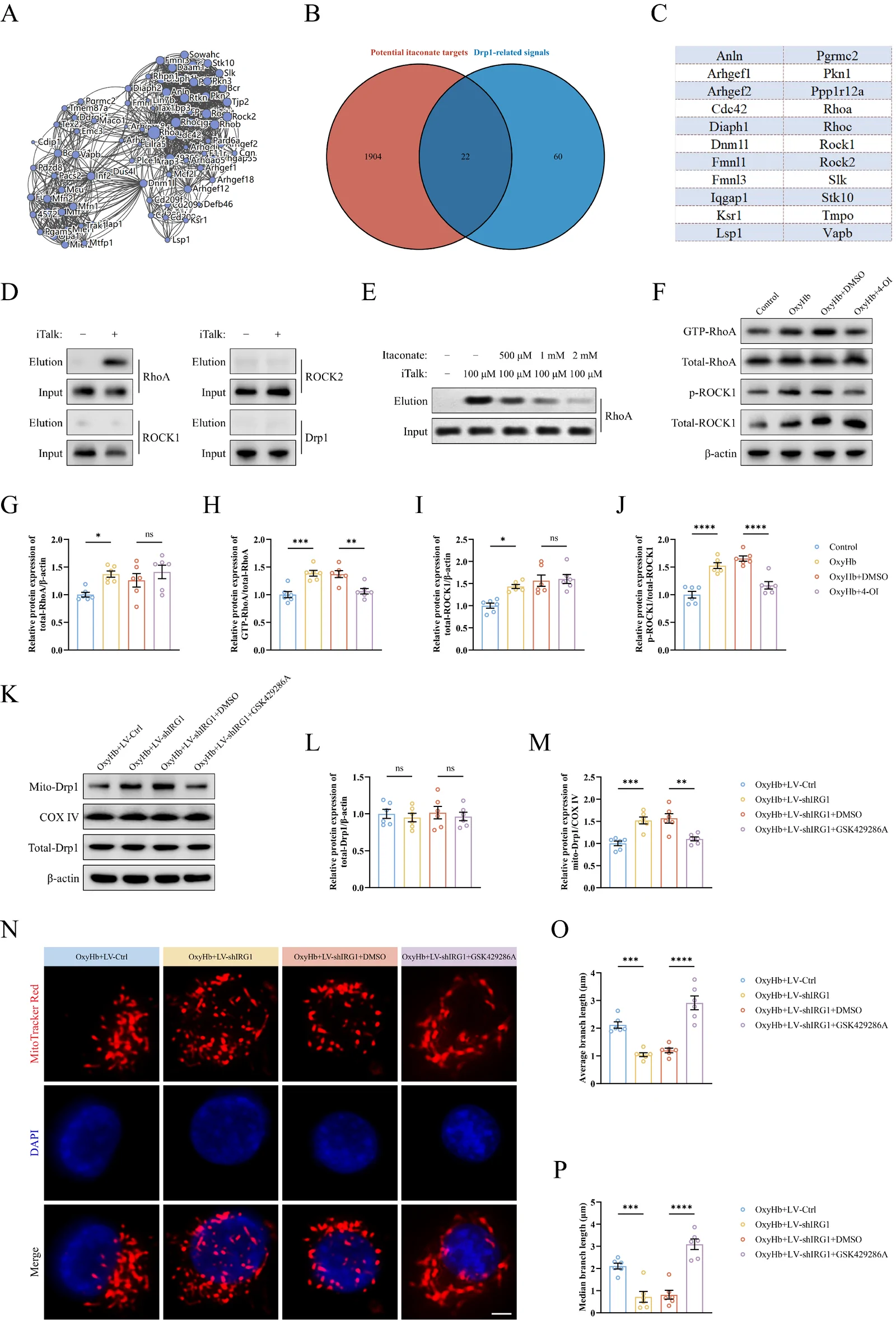
**The regulatory role of IRG1/itaconate axis in Drp1-dependent mitochondrial fission is mediated by RhoA/ROCK1 signaling**. (**A**) Construction of the PPI network of Drp1. (**B-C**) Intersection of itaconate cysteine alkylation targets and Drp1-related proteins from the PPI network. (**D**) iTalk probe and western blot to evaluate the interactions between itaconate and RhoA, ROCK1, ROCK2, and Drp1. (**E**) Competition experiment for the specific binding of iTalk probe to RhoA. (**F-J**) Western blot to evaluate the activation of RhoA/ROCK1 pathway in BV2 cells treated with Control, OxyHb, OxyHb+DMSO, and OxyHb+4-OI (n=6 independent biological replicates per group). (**K-M**) Western blot to evaluate the mitochondrial translocation of Drp1 in BV2 cells treated with OxyHb+LV-Ctrl, OxyHb+LV-shIRG1, OxyHb+LV-shIRG1+DMSO, and OxyHb+LV-shIRG1+GSK429286A (n=6 independent biological replicates per group). (**N-P**) Mito-Tracker Red CMXRos fluorescent probe to evaluate mitochondrial mean and median branch lengths (scale bar=5μm; n=6 independent biological replicates per group). ﻿Mitochondrial morphology was analyzed using the ImageJ mitochondrial morphology macro. Band intensities were quantified using ImageJ and normalized to the loading control. Image analysis was performed in a blinded manner. Data are presented as mean ± SEM, normalized to the first group, and analyzed by One-way ANOVA (G-J, L-M, O-P).

### Inhibition of RhoA/ROCK1 signaling rescues the exacerbation of microglial senescence-like transition and brain injury caused by IRG1 deficiency

We next investigated whether the RhoA/ROCK1 pathway mediates the effects of IRG1 deficiency on microglial senescence and brain injury. qPCR analysis demonstrated that the ROCK1 inhibitor GSK429286A rescued the upregulation of senescence markers (CCL5, CCL24, p16, and S100A8) in IRG1-deficient microglia following SAH (Fig. 6A-D). Additionally, GSK429286A reversed the exacerbated inflammatory cytokine expression, microglial proliferation, and pro-inflammatory polarization, while promoting the expression of the anti-inflammatory cytokine IL-10 (Fig. 6E-K; Supplementary Fig. 9A-D).

Moreover, GSK429286A treatment alleviated the worsened neurological deficits and brain edema in microglia-specific IRG1 cKO mice (Supplementary Fig. 9E-H). Furthermore, it attenuated the increased neuronal death and degeneration induced by IRG1 knockout (Fig. 6L-O). These findings indicate that IRG1 deficiency promotes microglial senescence and neuroinflammation via the RhoA/ROCK1 pathway, and that the IRG1/itaconate axis exerts its neuroprotective effects by inhibiting this signaling cascade.

### Itaconate inhibits RhoA/ROCK1 signaling activation by alkylating the C107 site of RhoA

To identify the specific cysteine residues on RhoA modified by itaconate, we overexpressed RhoA in 293T cells and incubated them with 1 mM itaconate. Mass spectrometry analysis of enriched RhoA revealed a mass shift corresponding to itaconate modification (+130.10 Da, C5H6O4) at cysteine residues C16, C107, and C159 (Fig. 7A-C). Similarly, incubation with 250 μM 4-OI resulted in a mass shift consistent with 4-OI modification (+242.1518 Da, C13H22O4) at the same three sites (Fig. 7D-F).

To determine the functional significance of these sites, we generated RhoA mutants (C16S, C107S, and C159S) and expressed them in OxyHb-stimulated BV2 cells. Among the mutants, only C107S abolished the inhibitory effect of 4-OI on RhoA activation (Fig. 7G-H). Crucially, the C107S mutation also reversed the 4-OI-mediated suppression of the RhoA/ROCK1 signaling pathway (Fig. 7I-K). Furthermore, multiple sequence alignment of RhoA across diverse species—including *Homo sapiens*, *Mus musculus*, *Rattus norvegicus*, *Pan troglodytes*, *Bos taurus*, *Canis lupus familiaris*, *Sus scrofa*, *Danio rerio*, *Oryctolagus cuniculus*, and *Caenorhabditis elegans*—revealed that the C107 residue is highly conserved (Supplementary Fig. 10A), suggesting its evolutionary importance and the potential generalizability of our findings across models. These results demonstrate that itaconate inhibits RhoA/ROCK1 pathway activation specifically by alkylating the C107 residue of RhoA.

### C107S mutation of RhoA reverses the inhibitory effect of itaconate derivative on Drp1-dependent mitochondrial fission and senescence-like transition

Finally, we examined whether alkylation at RhoA-C107 is required for the protective effects of itaconate against Drp1-mediated fission and microglial senescence. Western blot analysis showed that the ability of 4-OI to inhibit Drp1 mitochondrial translocation was lost in cells expressing the RhoA-C107S mutant (Fig. 8A-C). Mito-Tracker staining confirmed that the C107S mutation abolished the reduction in mitochondrial fission induced by 4-OI (Fig. 8D-F). Furthermore, the C107S mutation negated the protective effects of 4-OI on mitochondrial membrane potential, permeability, and oxidative stress (Fig. 8G-J).

Consistent with these findings, qPCR analysis revealed that the C107S mutation abolished the 4-OI-mediated suppression of senescence markers (CCL5, CCL24, and S100A8) (Supplementary Fig. 11A-C) and inflammatory mediators (IL-1β, IL-6, TNF-α, COX-2, and iNOS) (Supplementary Fig. 11D-E; Supplementary Fig. 11G-I). Although 4-OI still exerted some inhibitory effect on IL-18 expression in the C107S mutant, the effect was significantly attenuated compared to wild-type cells (Supplementary Fig. 11F). Collectively, these data provide conclusive evidence that itaconate inhibits the RhoA/ROCK1 pathway via alkylation of RhoA at C107, thereby suppressing Drp1-mediated mitochondrial fission, microglial senescence-like transition, and neuroinflammation following SAH.

**Figure 6.**
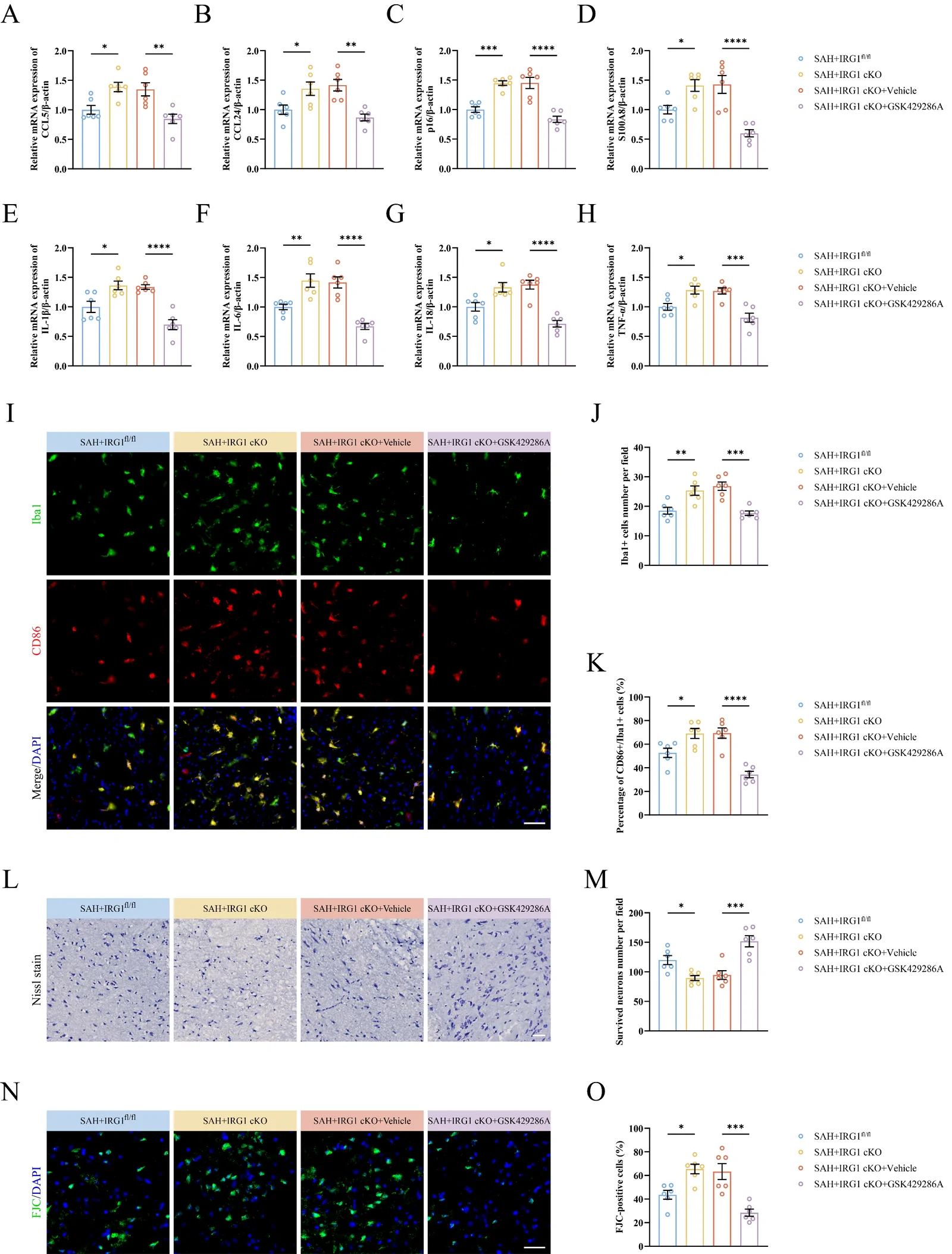
**GSK429286A rescues the exacerbation of microglial senescence-like transition and brain injury caused by IRG1 deficiency**. (**A-H**) qPCR to evaluate CCL5, CCL24, p16, S100A8, IL-1β, IL-6, IL-18, and TNF-α expressions in mice of SAH+IRG1^fl/fl^, SAH+IRG1 cKO, SAH+IRG1 cKO+Vehicle, and SAH+IRG1 cKO+GSK429286A groups (n=6 mice per group). (**I-K**) Immunofluorescence staining to evaluate microglial number and polarization (scale bar=60μm; n=6 mice per group). (**L-M**) Nissl staining to assess neuronal survival (scale bar=40μm; n=6 mice per group). (**N-O**) FJC staining to assess neuronal degeneration and death (scale bar=40μm; n=6 mice per group). Relative mRNA levels were calculated using the 2^-ΔΔCt^ method. Image analysis was performed in a blinded manner. Data are presented as mean ± SEM, normalized to the first group, and analyzed by One-way ANOVA (A-H, J-K, M, O).

**Figure 7.**
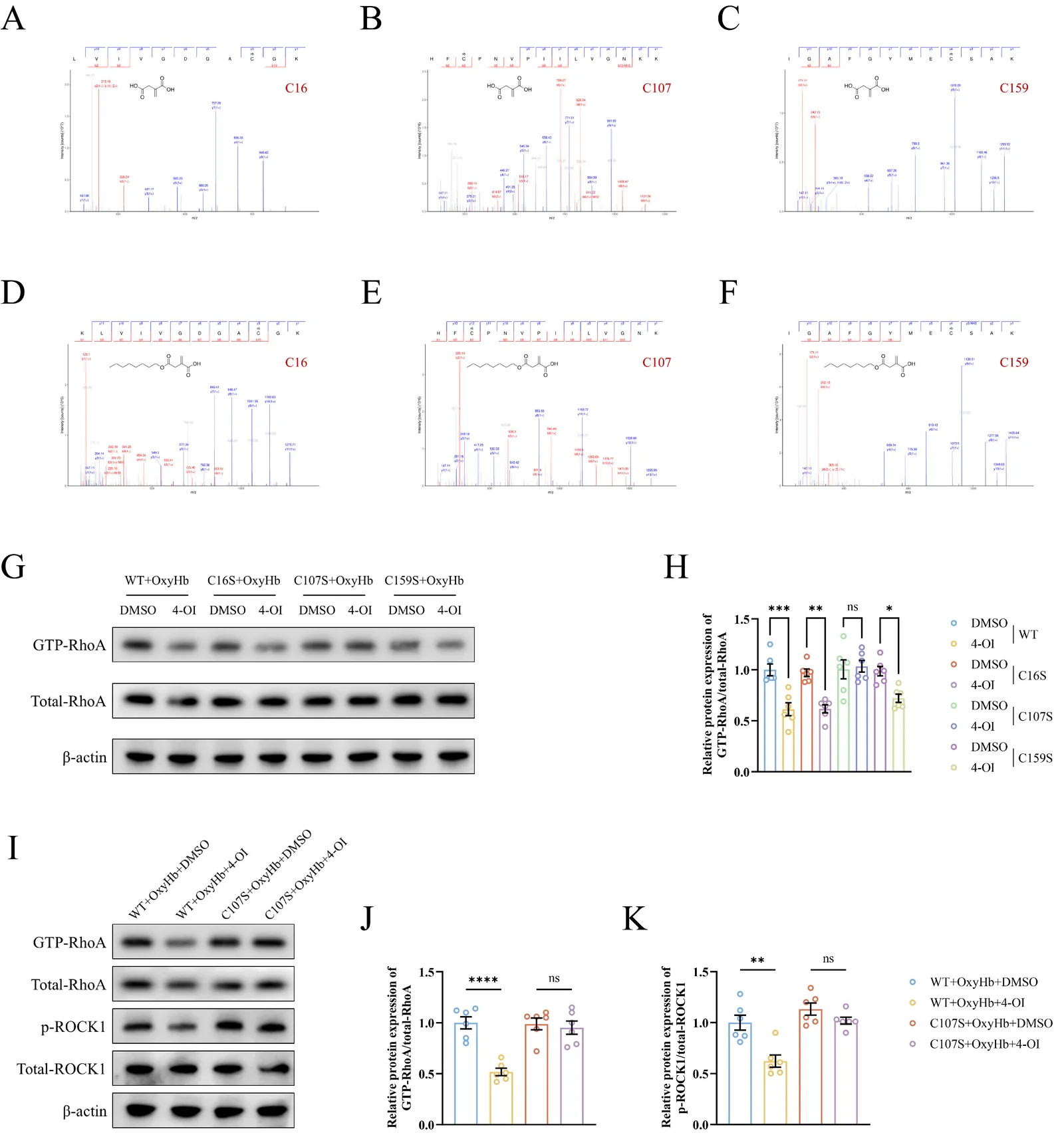
**C107S mutation of RhoA reverses the inhibitory effect of 4-OI on RhoA/ROCK1 signaling activation**. (**A-C**) Mass spectrometry analysis showing the alkylation of RhoA at C16, C107, and C159 by itaconate (C5H6O4). (**D-F**) Mass spectrometry analysis showing the alkylation of RhoA at C16, C107, and C159 by 4-OI (C13H22O4). (**G-H**) Western blot to evaluate RhoA activation in BV2 cells of WT+DMSO, WT+4-OI, C16S+DMSO, C16S+4-OI, C107S+DMSO, C107S+4-OI, C159S+DMSO, and C159S+4-OI groups (n=6 independent biological replicates per group). (**I-K**) Western blot to evaluate the activation of RhoA/ROCK1 pathway in BV2 cells of WT+OxyHb+DMSO, WT+OxyHb+4-OI, C107S+OxyHb+DMSO, and C107S+OxyHb+4-OI groups (n=6 independent biological replicates per group). Band intensities were quantified using ImageJ and normalized to the loading control. Data are presented as mean ± SEM, normalized to the first group, and analyzed by One-way ANOVA (H, J-K).

## DISCUSSION

In the present study, we unveil a critical immunometabolic mechanism underlying the age-related exacerbation of EBI following SAH. We demonstrate that the IRG1/itaconate axis functions as a vital endogenous checkpoint that restrains the senescence-like transition of microglia. Specifically, we found that the protective upregulation of IRG1 and subsequent itaconate production are significantly blunted in the aged brain following SAH. Mechanistically, we identified that itaconate exerts its neuroprotective effects by directly alkylating the small GTPase RhoA at the C107 residue. This post-translational modification inhibits RhoA-GTP binding and downstream ROCK1 activation, thereby suppressing Drp1-mediated mitochondrial fission and preventing the acquisition of a SASP (Fig. 9). These findings not only elucidate a novel metabolic-mitochondrial signaling axis (IRG1/itaconate-RhoA-Drp1) but also suggest that restoring this pathway represents a promising therapeutic strategy for elderly SAH patients.

**Figure 8.**
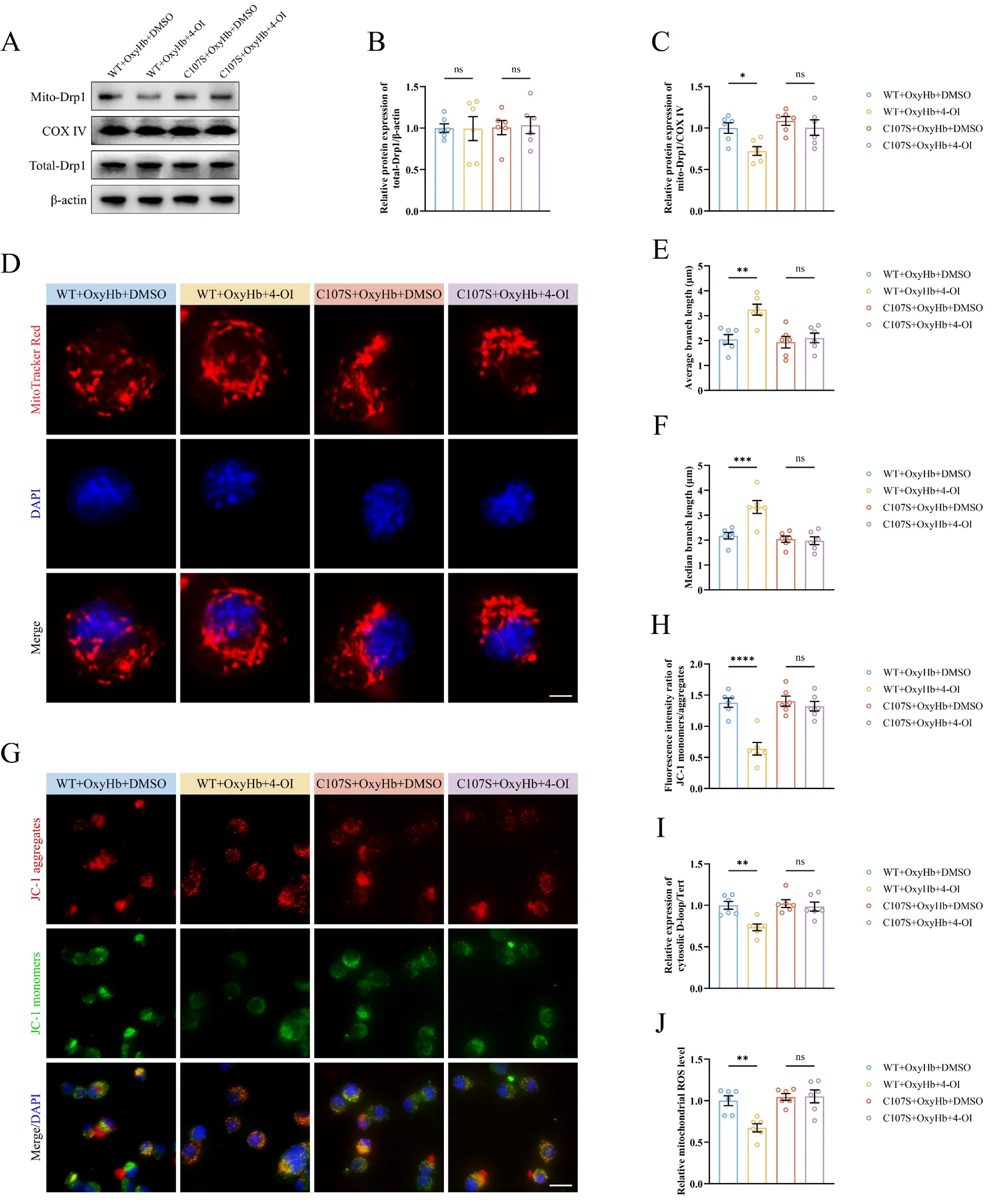
**C107S mutation of RhoA reverses the inhibitory effect of 4-OI on Drp1-dependent mitochondrial fission and its improving effect on mitochondrial function**. (**A-C**) Western blot to evaluate the mitochondrial translocation of Drp1 in BV2 cells of WT+OxyHb+DMSO, WT+OxyHb+4-OI, C107S+OxyHb+DMSO, and C107S+OxyHb+4-OI groups (n=6 independent biological replicates per group). (**D-F**) Mito-Tracker Red CMXRos fluorescent probe to evaluate mitochondrial mean and median branch lengths (scale bar=5μm; n=6 independent biological replicates per group). ﻿Mitochondrial morphology was analyzed using the ImageJ mitochondrial morphology macro. (**G-H**) JC-1 fluorescent probe to evaluate mitochondrial membrane potential level (scale bar=20μm; n=6 independent biological replicates per group). Fluorescence ratios (Green/Red) were calculated to determine membrane potential. (**I**) qPCR to evaluate cytoplasmic D-loop level (n=6 independent biological replicates per group). (**J**) MitoSOX Red to evaluate mitochondrial ROS level (n=6 independent biological replicates per group). Band intensities were quantified using ImageJ and normalized to the loading control. Image analysis was performed in a blinded manner. Data are presented as mean ± SEM, normalized to the first group, and analyzed by One-way ANOVA (B-C, E-F, H-J).

The impact of aging on SAH prognosis is profound [3, 6], yet the molecular underpinnings remain elusive. Our data reveal that while basal levels of IRG1 are elevated in the aged brain—likely reflecting a state of chronic low-grade inflammation or inflammaging—the capacity to further upregulate this metabolic brake in response to acute hemorrhagic stress is severely compromised. This blunted response aligns with the concept of immunosenescence, where immune cells lose their plasticity and ability to mount appropriate responses to new insults [43, 44]. IRG1, as the rate-limiting enzyme for itaconate synthesis, has been established as a negative regulator of inflammation in macrophages [45-48]. Our findings extend this to the CNS, suggesting that the failure to robustly engage the IRG1/itaconate axis renders aged microglia susceptible to dysregulated activation and senescence-like transition, thereby amplifying neuroinflammation and secondary brain injury.

A key conceptual advance of our study is the characterization of the microglial senescence-like transition as a driver of SAH pathology. Unlike replicative senescence caused by telomere shortening, this stress-induced premature senescence is characterized by cell cycle arrest and the robust secretion of pro-inflammatory mediators (SASP), which perpetuate a toxic microenvironment [49-51]. We demonstrate that mitochondrial dynamics are central to this phenotypic switch. Mitochondrial dysfunction, particularly the shift towards excessive fission, is a hallmark of cellular senescence [52-54]. Our results show that SAH induces Drp1-dependent mitochondrial fragmentation, leading to ROS accumulation and mtDNA release. Importantly, we provide evidence that the IRG1/itaconate axis preserves mitochondrial homeostasis by restraining Drp1 hyperactivity. This is consistent with recent reports linking mitochondrial dynamics to microglial activation states [55, 56], but uniquely positions itaconate as an upstream metabolic regulator of this morphological machinery.

**Figure 9.**
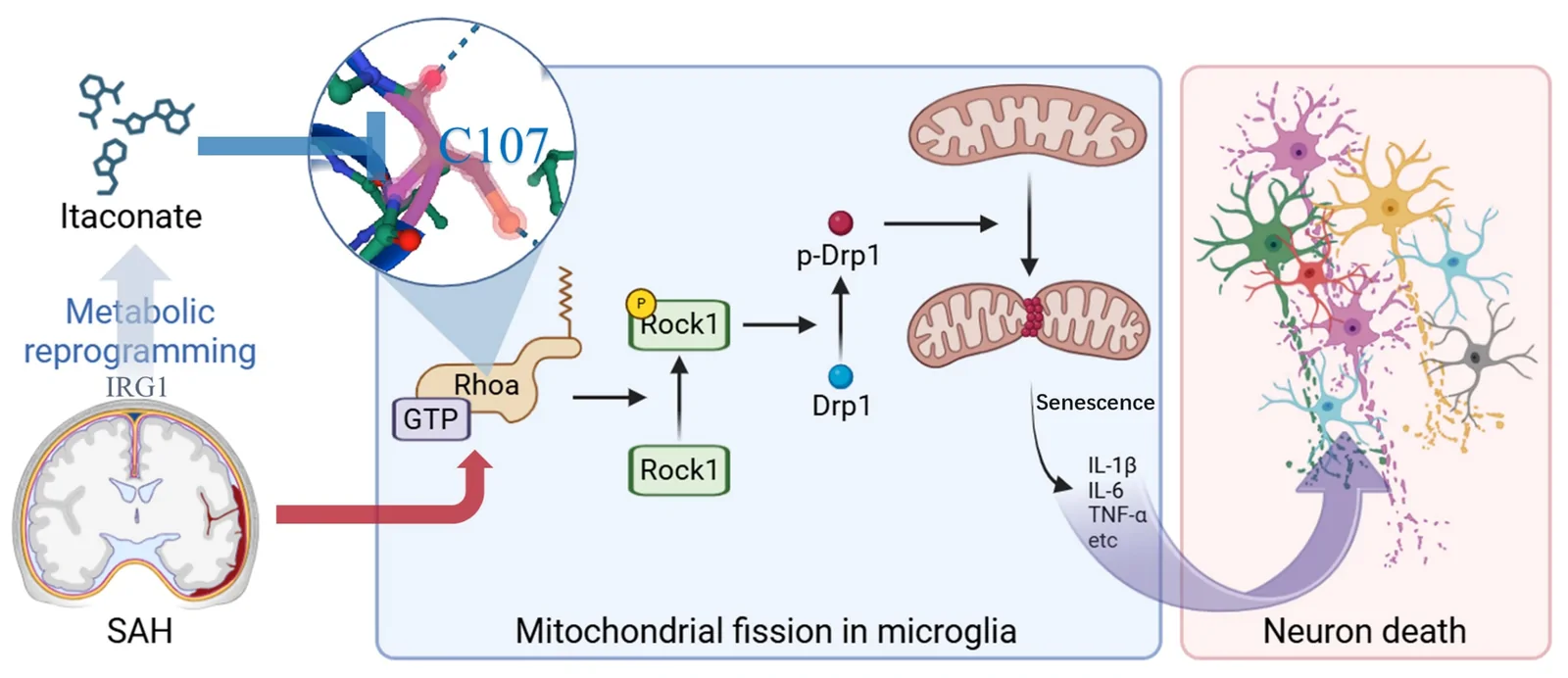
**Schematic representation by which IRG1/itaconate exerts neuroprotective effects through alkylating RhoA to inhibit microglial senescence-like transition in SAH**. Itaconate alkylates RhoA at cysteine 107 to inhibit the RhoA/ROCK1/Drp1 signaling cascade. This inhibition prevents excessive mitochondrial fission and the senescence-like transition of microglia, thereby attenuating neuronal death and brain injury in SAH.

The most significant mechanistic discovery of this work is the identification of RhoA as a direct target of itaconate alkylation. Itaconate is an electrophilic metabolite known to modify cysteine residues on proteins such as KEAP1, NLRP3, and GAPDH via Michael addition [30, 31, 57]. Specifically, itaconate-mediated alkylation of cysteine 151, 257, 288, 273 and 297 on KEAP1 triggers the Nrf2-dependent antioxidant response [30], while its modification of cysteine 548 on NLRP3 disrupts inflammasome assembly to limit excessive inflammation [31]. Furthermore, itaconate targets GAPDH to suppress aerobic glycolysis, thereby facilitating metabolic rewiring in activated macrophages [57]. Here, using click chemistry and mass spectrometry, we identified C107 of RhoA as a specific site for itaconate modification. Our mutagenesis experiments confirmed that alkylation at this site prevents RhoA activation, subsequently blocking the RhoA/ROCK1 signaling cascade. Notably, the significance of Cys107 as a regulatory target is supported by recent structural studies. It has been reported that Cys107 resides within a conserved allosteric groove, and its covalent modification by small-molecule inhibitors (e.g., DC-Rhoin) can disrupt the interaction between RhoA and its regulatory proteins, such as GEFs and GDIs [58]. This suggests that C107 is a critical node for both endogenous metabolic control and pharmacological intervention. While other residues like Cys16—located near the nucleotide-binding pocket—have also been identified as targets for covalent inhibition to block GTP binding [59], our findings uniquely define C107 as a key site for itaconate-mediated immunometabolic regulation.

The RhoA/ROCK pathway is a well-known regulator of cytoskeletal reorganization, but its role in mitochondrial dynamics is gaining attention [60, 61]. We show that ROCK1, activated by RhoA, promotes Drp1 phosphorylation at Ser616, a prerequisite for Drp1 mitochondrial translocation and fission [62, 63]. By alkylating RhoA, itaconate effectively locks this pathway, preventing the mitochondrial fragmentation that drives the senescence-like transition. This defines a precise molecular link between immunometabolism and organelle dynamics.

While our study demonstrates that itaconate successfully alkylates RhoA, the precise regulatory mechanisms governing this post-translational modification warrant further investigation. Structurally, itaconate is an α,β-unsaturated dicarboxylic acid that acts as a Michael acceptor, suggesting that the alkylation of RhoA is likely a spontaneous, non-enzymatic reaction driven by the local accumulation of itaconate and the specific pKa of the targeted cysteine thiolate [30, 64]. However, the potential for enzymatic facilitation cannot be entirely excluded [65]. Furthermore, the reversibility of this modification remains an open question. It is currently unknown whether RhoA dealkylation occurs spontaneously via a retro-Michael reaction [29], or if it requires a yet-unidentified deitaconase enzyme. Alternatively, if the thioether bond formed during itaconation is highly stable in the cellular environment, the restoration of the unmodified RhoA pool may depend not on direct dealkylation, but rather on the proteasomal degradation of the modified protein and subsequent *de novo* protein synthesis [66]. Clarifying the kinetic stability and potential enzymatic regulation of this alkylation will be critical for understanding how itaconate dynamically modulates RhoA signaling during the inflammatory response.

Additionally, our findings provide a crucial mechanistic link between metabolic reprogramming and the sterile inflammation characteristic of SAH. In activated immune cells, the break in the TCA cycle at the IDH step directs carbon flux towards IRG1 for itaconate production, a process often viewed primarily as a metabolic shift from mitochondrial respiration to glycolysis [27, 45]. However, our data suggest that this axis also serves a vital structural function by maintaining organelle integrity. The leakage of mtDNA into the cytosol, which we observed to be significantly exacerbated by IRG1 deficiency, acts as a potent damage-associated molecular pattern (DAMP) [67]. While we focused on the upstream regulation of fission, cytosolic mtDNA is a well-established trigger for cytosolic DNA sensors, such as the cGAS-STING pathway and the NLRP3 inflammasome, which are the primary drivers of the SASP [68, 69]. Therefore, by alkylating RhoA and curbing Drp1-mediated fission, itaconate effectively limits this mitochondrial DAMP release. This implies that the IRG1/itaconate axis functions not merely as a metabolic byproduct, but as an active mitochondrial gatekeeper that prevents the acute metabolic stress of SAH from escalating into a chronic, self-perpetuating cycle of senescence and neuroinflammation.

Our study has important clinical implications. Current pharmacological interventions for SAH are limited, especially for elderly patients who suffer from poorer outcomes [3, 6]. Notably, Fasudil, a potent ROCK inhibitor, has been clinically utilized to alleviate cerebral vasospasm and improve outcomes in SAH patients [70]. Our discovery that itaconate acts as an endogenous inhibitor of the RhoA/ROCK1 signaling pathway via RhoA alkylation provides a novel metabolic rationale for the clinical efficacy of ROCK-targeted therapies. Unlike the competitive inhibition of ROCK by Fasudil, itaconate functions as an upstream metabolic brake that prevents the initial activation of the RhoA/ROCK cascade. This suggests that restoring the IRG1/itaconate axis—perhaps through 4-OI or similar derivatives—could complement current Fasudil-based treatments or serve as a more targeted metabolic intervention to suppress microglial senescence. The efficacy of the cell-permeable itaconate derivative, 4-OI, in rescuing the phenotype of IRG1-deficient microglia and alleviating brain injury in our model highlights the translational potential of itaconate-based therapeutics. By targeting the metabolic reprogramming of microglia, rather than merely blocking downstream cytokines, 4-OI may offer a more comprehensive resolution of neuroinflammation. Furthermore, the identification of the RhoA-C107 site provides a specific structural target for future drug design, potentially allowing for the development of small molecules that mimic the alkylating effect of itaconate with higher specificity.

Furthermore, while our study provides robust evidence in murine models, the translational potential of the IRG1/itaconate axis to human SAH necessitates a nuanced consideration of interspecies differences. It is well-established that human and murine microglia exhibit divergent transcriptional profiles, particularly concerning aging-related genes and metabolic signatures [71]. For instance, while the induction of IRG1 is a conserved feature of activated myeloid cells, the magnitude and kinetics of itaconate production may vary between species due to differences in basal metabolic rates and the microglial priming state in the aged human brain [72]. Nevertheless, several factors bolster the clinical relevance of our findings. First, the Cys107 residue of RhoA, which we identified as the primary site for itaconate-mediated alkylation, is 100% conserved between *Mus musculus* and *Homo sapiens*, suggesting that this metabolic-mitochondrial check is a fundamental evolutionary mechanism. Second, human post-mortem studies have confirmed that aged microglia undergo a senescence-like transition characterized by a SASP profile similar to what we observed in mice [73, 74]. Therefore, although the precise metabolic flux may differ, the downstream targeting of the RhoA/ROCK1/Drp1 axis likely remains a viable therapeutic strategy for human SAH patients.

Despite these findings, our study has limitations. First, because Cx3cr1 is also expressed in peripheral macrophages and monocytes, our Cx3cr1-Cre model is not exclusively microglia-specific. Thus, IRG1 expression and itaconate production may partially stem from infiltrating myeloid cells, contributing to the overall neuroprotective effect. Second, while we utilized 10-month-old mice to model the clinical reality of SAH, we acknowledge that this age represents a relatively early stage of aging, which may not fully capture the profound senescent changes seen in much older cohorts. Furthermore, the complex comorbidities frequently observed in elderly SAH patients, such as hypertension or diabetes, were not incorporated into our current model. As these factors significantly influence the clinical progression and prognosis of SAH, the lack of comorbid models may limit the direct translatability of our findings. Future studies should, therefore, investigate the efficacy of itaconate derivatives in both older mice and comorbid models to more accurately mimic the clinical complexities of the geriatric population. Additionally, although we have included supplementary data from female mice to address potential sex differences, our mechanistic investigations and long-term functional assessments were primarily focused on male cohorts. Given the known sexual dimorphism in microglial response and SAH pathophysiology, further comprehensive studies are required to determine if the IRG1/itaconate axis operates through identical molecular pathways in both sexes. Finally, while we identified RhoA as a key target, the broad electrophilic nature of itaconate implies it may modify other proteins involved in mitochondrial dynamics or senescence, warranting further proteomic exploration.

In conclusion, our study identifies the IRG1/itaconate axis as a critical endogenous defense mechanism that is impaired by aging following SAH. We elucidate a novel pathway wherein itaconate alkylates RhoA at C107 to inhibit the RhoA/ROCK1/Drp1 cascade, thereby preventing excessive mitochondrial fission and the microglial senescence-like transition. These findings provide a mechanistic rationale for targeting immunometabolism and mitochondrial dynamics to improve outcomes in SAH, particularly in the vulnerable elderly population.

## Data Availability

The proteomics data analyzed in this study were derived from a previously published study and are publicly available at https://pubs.acs.org/doi/10.1021/jacs.9b11962. Supplementary Supplementary Tables 3-5 are available in the Dryad repository (https://datadryad.org/) under the identifier DOI: 10.5061/dryad.9cnp5hr0x. All other data supporting the findings of this study are available from the corresponding authors upon reasonable request.

## Supplementary Materials

The Supplementary data are available online at: www.aginganddisease.org/EN/10.14336/AD.2025.1527.
