# Supplementary material for: IRG1/Itaconate Inhibits Microglial Senescence-Like Transition by Modulating Mitochondrial Dynamics through Rhoa Alkylation in Subarachnoid Hemorrhage
Source: Aging Dis. 2026 Apr 1;17(6):3514–36. doi: 10.14336/AD.2025.1527 (PMC13653722; doi:10.14336/AD.2025.1527)
Supplement: Supplementary file 1 [file AD-17-6-3514-s.pdf]

SUPPLEMENTARY DATA

IRG1/Itaconate Inhibits Microglial Senescence-Like Transition by Modulating Mitochondrial Dynamics through Rhoa Alkylation in Subarachnoid Hemorrhage

Zeyu Zhang, Yanlin Chen, Weizhen Gao, Fengchen Zhang, Yichao Jin, Jie Li, Jiaqi Wang, Tao Lv, Feng Jia, Xiaohua Zhang

© 2026. Zhang Z et al. Published online at http://www.aginganddisease.org/EN/10.14336/AD.2025.1527

SUPPLEMENTARY DATA

Supplementary Figures

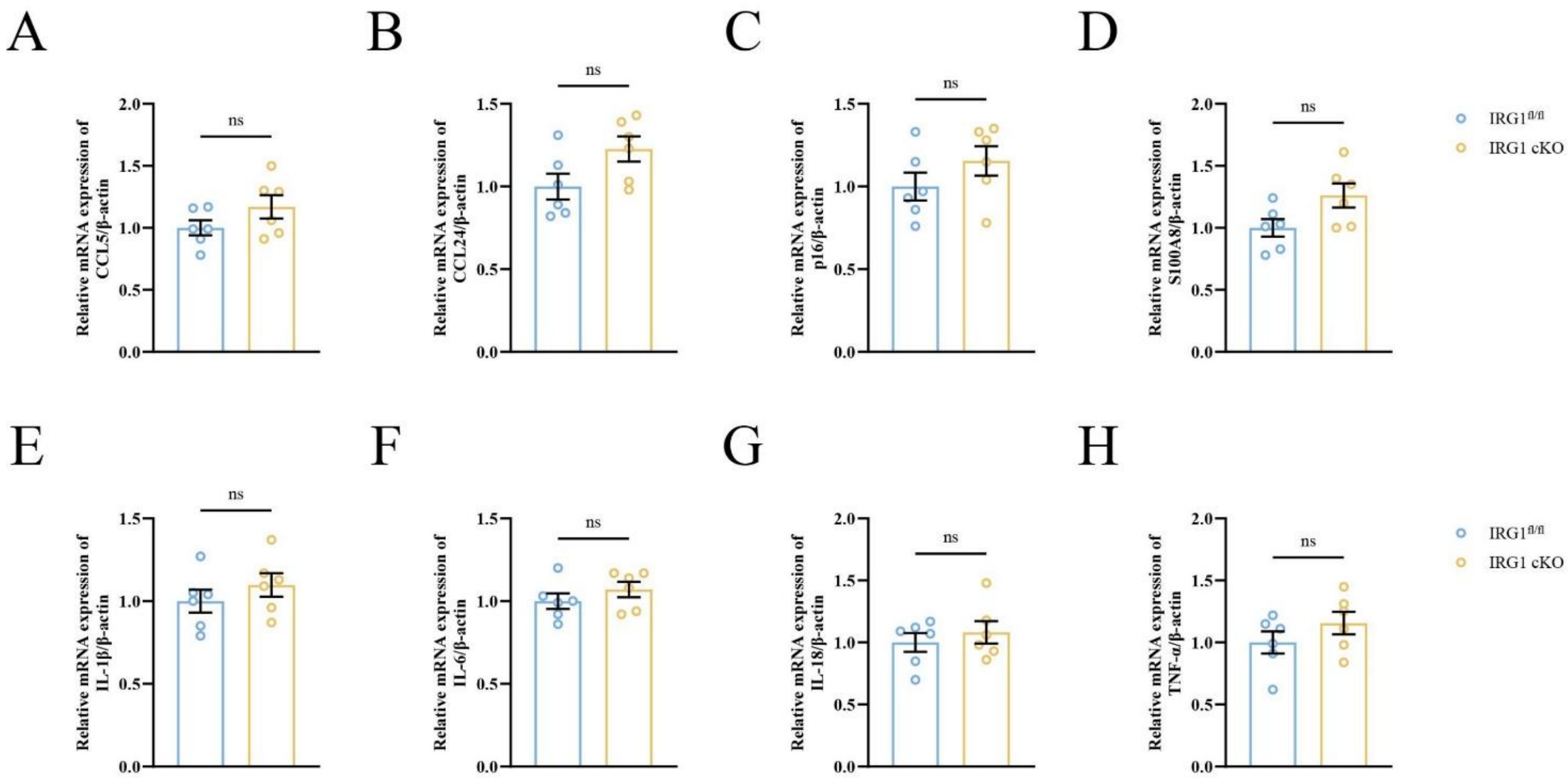

Supplementary Fig. 1. Effect of IRG1 deficiency on microglial senescence-like transition under physiological conditions. A-H. qPCR to evaluate CCL5, CCL24, p16, S100A8, IL-1β, IL-6, IL-18, and TNF-α expressions in mice of $IRG1^{fl/fl}$ and IRG1 cKO groups (n=6 mice per group). Relative mRNA levels were calculated using the $2^{-\Delta\Delta Ct}$ method. Data are presented as mean ± SEM, normalized to the first group, and analyzed by Student's t-test (**A-H**).

© 2026. Zhang Z et al. Published online at http://www.aginganddisease.org/EN/10.14336/AD.2025.1527

SUPPLEMENTARY DATA

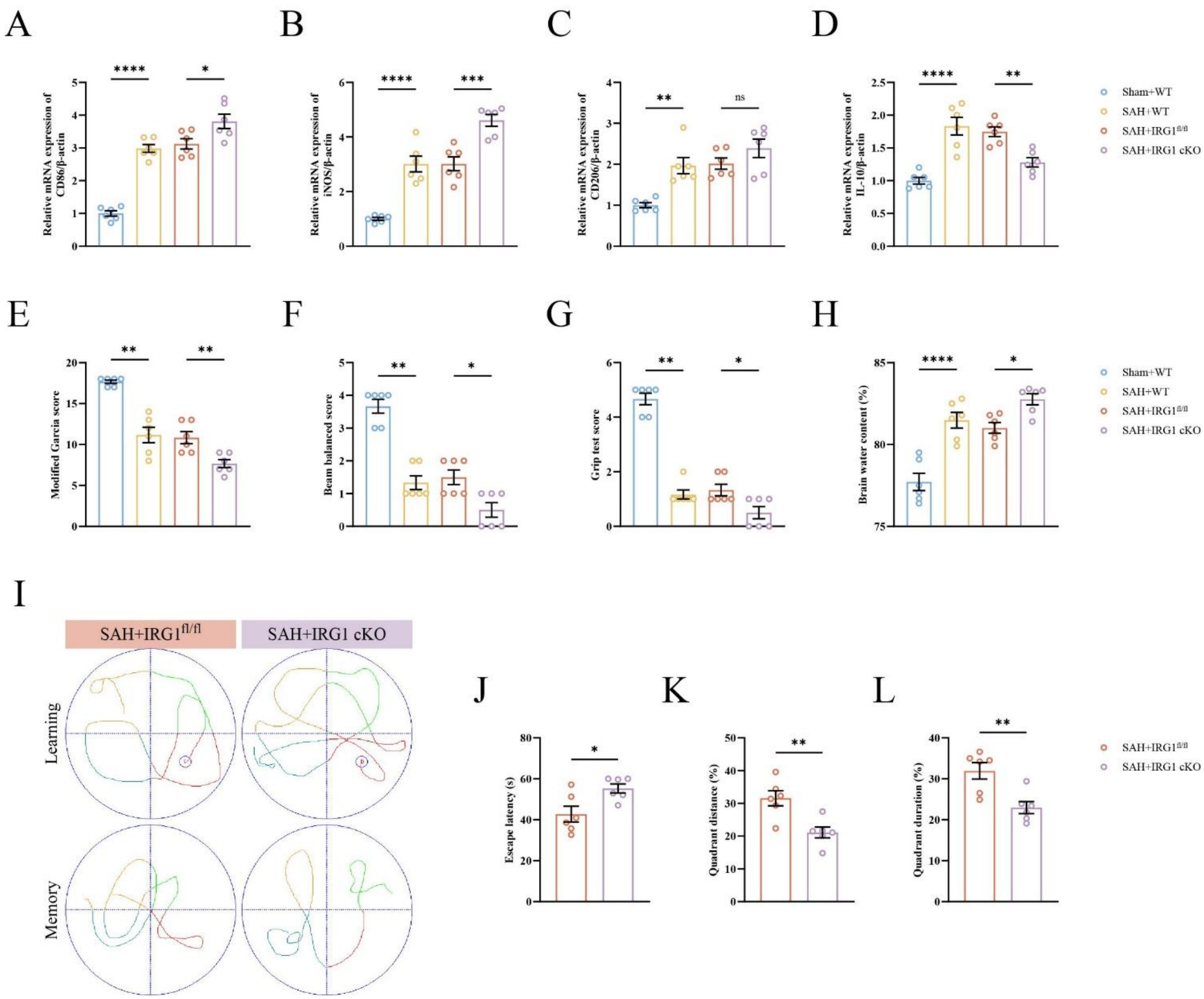

Supplementary Fig. 2. IRG1 deficiency exacerbates microglial inflammatory activation and neurological deficits after SAH. **A-D.** qPCR to evaluate CD86, iNOS, CD206, and IL-10 expressions in mice of Sham+WT, SAH+WT, SAH+IRG1$^{fl/fl}$, and SAH+IRG1 cKO groups (n=6 mice per group). **E-G.** Short-term neurological function at 24 h post-SAH (n=6 mice per group). Assessments include the modified Garcia score (evaluating 6 parameters: activity, symmetry, extension, climbing, proprioception, and reflex), beam balance, and grip tests. Higher scores indicate superior neurological performance. **H.** Measurement of brain water content (n=6 mice per group). **I-L.** Long-term neurological function from days 22–26 post-SAH in mice of SAH+IRG1$^{fl/fl}$ and SAH+IRG1 cKO groups (n=6 mice per group). Spatial learning was assessed via escape latency in the Morris water maze (days 22–25). Memory retention was evaluated on day 26 via a probe trial, recording swim trajectories and the percentage of time and distance spent in the target quadrant. Relative mRNA levels were calculated using the $2^{-\Delta\Delta Ct}$ method. All behavioral assessments were performed by two investigators blinded to the experimental groups. Data are presented as mean ± SEM, normalized to the first group, and analyzed by One-way ANOVA (**A-D**, **H**), Kruskal-Wallis H test (**E-G**), or Student's t-test (**J-L**).

© 2026. Zhang Z et al. Published online at http://www.aginganddisease.org/EN/10.14336/AD.2025.1527

SUPPLEMENTARY DATA

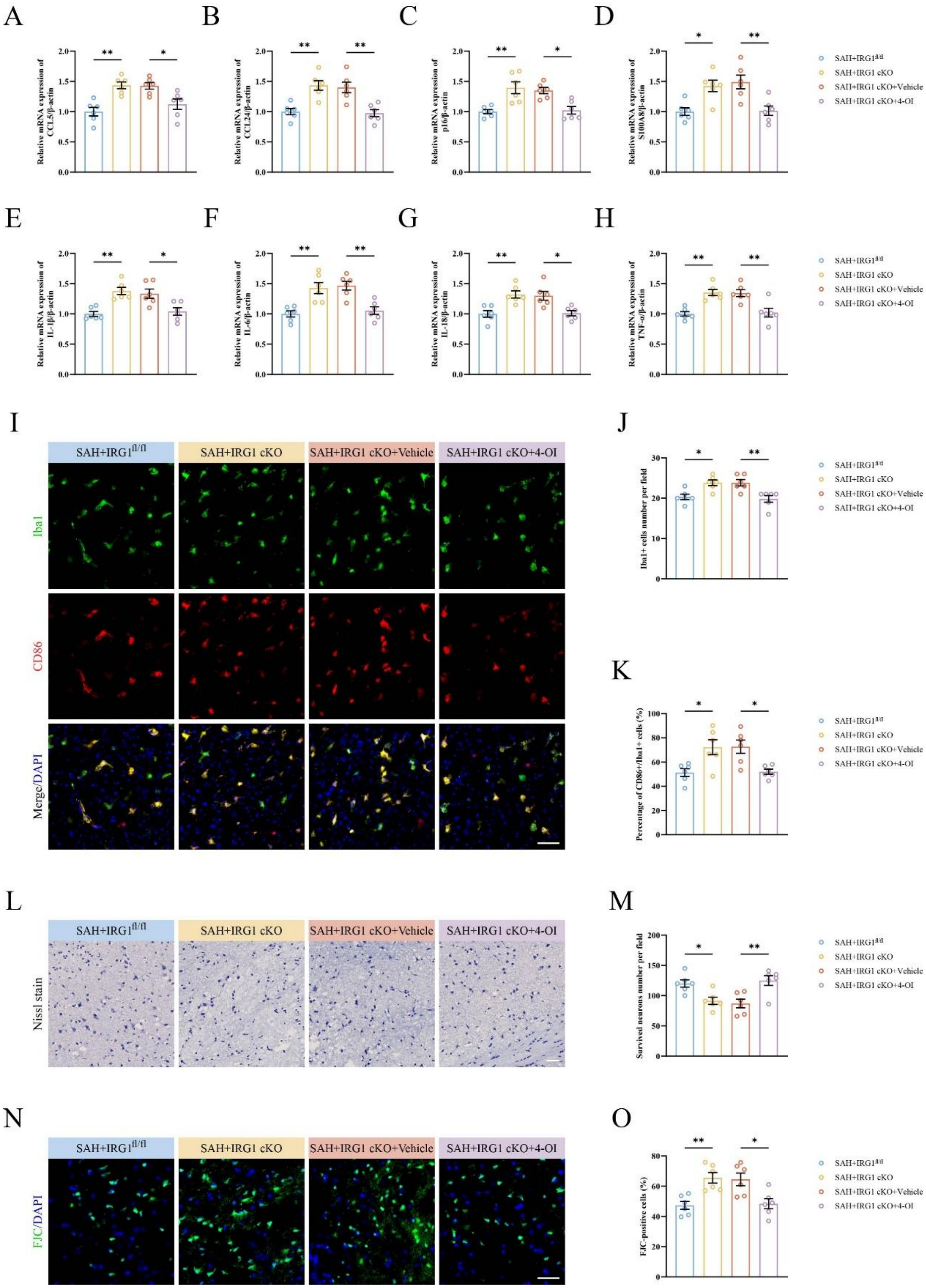

Supplementary Fig. 3. 4-OI rescues the exacerbation of microglial senescence-like transition and brain injury caused by IRG1 deficiency. A-H. qPCR to evaluate CCL5, CCL24, p16, S100A8, IL-1β, IL-6, IL-18, and TNF-α expressions in mice of SAH+IRG1$^{fl/fl}$, SAH+IRG1 cKO, SAH+IRG1 cKO+Vehicle, and SAH+IRG1 cKO+4-OI groups (n=6 mice per group). **I-K.** Immunofluorescence staining to evaluate microglial number and polarization (scale bar=60μm; n=6 mice per group). **L-M.** Nissl staining to assess neuronal survival (scale bar=40μm; n=6 mice per group). **N-O.** FJC staining to assess neuronal degeneration and death (scale bar=40μm; n=6 mice per group). Relative mRNA levels were calculated using the

© 2026. Zhang Z et al. Published online at http://www.aginganddisease.org/EN/10.14336/AD.2025.1527

$2^{-\Delta\Delta Ct}$ method. Image analysis was performed in a blinded manner. Data are presented as mean ± SEM, normalized to the first group, and analyzed by One-way ANOVA (**A-H**, **J-K**, **M**, **O**).

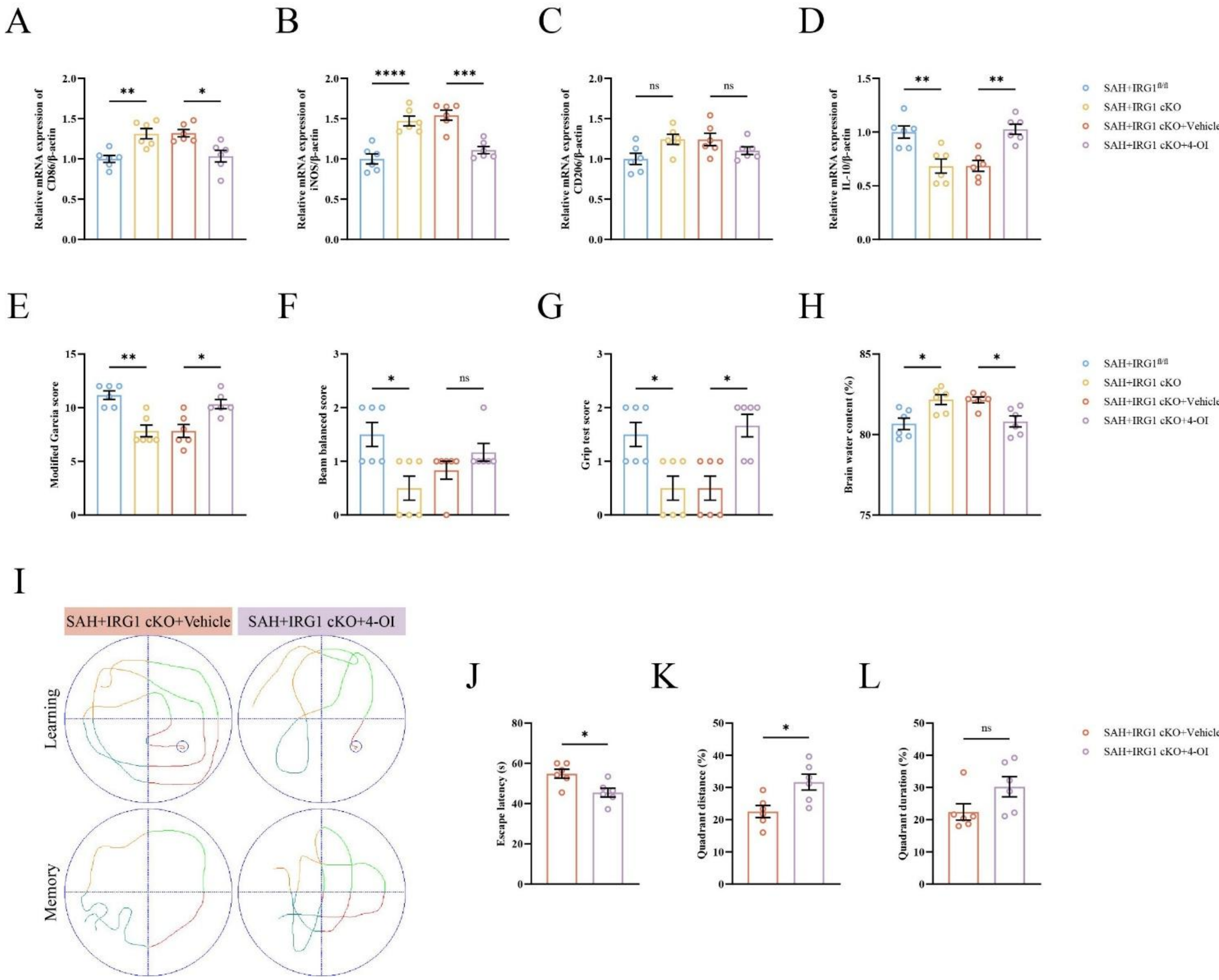

Supplementary Fig. 4. 4-OI rescues the exacerbation of microglial inflammatory activation and neurological deficits caused by IRG1 deficiency. **A-D.** qPCR to evaluate CD86, iNOS, CD206, and IL-10 expressions in mice of SAH+IRG1$^{fl/fl}$, SAH+IRG1 cKO, SAH+IRG1 cKO+Vehicle, and SAH+IRG1 cKO+4-OI groups (n=6 mice per group). **E-G.** Short-term neurological function at 24 h post-SAH (n=6 mice per group). Assessments include the modified Garcia score (evaluating 6 parameters: activity, symmetry, extension, climbing, proprioception, and reflex), beam balance, and grip tests. Higher scores indicate superior neurological performance. **H.** Measurement of brain water content (n=6 mice per group). **I-L.** Long-term neurological function from days 22–26 post-SAH in mice of SAH+IRG1 cKO+Vehicle and SAH+IRG1 cKO+4-OI groups (n=6 mice per group). Spatial learning was assessed via escape latency in the Morris water maze (days 22–25). Memory retention was evaluated on day 26 via a probe trial, recording swim trajectories and the percentage of time and distance spent in the target quadrant. Relative mRNA levels were calculated using the $2^{-\Delta\Delta Ct}$ method. All behavioral assessments were performed by two investigators blinded to the experimental groups. Data are presented as mean ± SEM, normalized to the first group, and analyzed by One-way ANOVA (**A-D**, **H**), Kruskal-Wallis H test (**E-G**), or Student's t-test (**J-L**).

© 2026. Zhang Z et al. Published online at http://www.aginganddisease.org/EN/10.14336/AD.2025.1527

SUPPLEMENTARY DATA

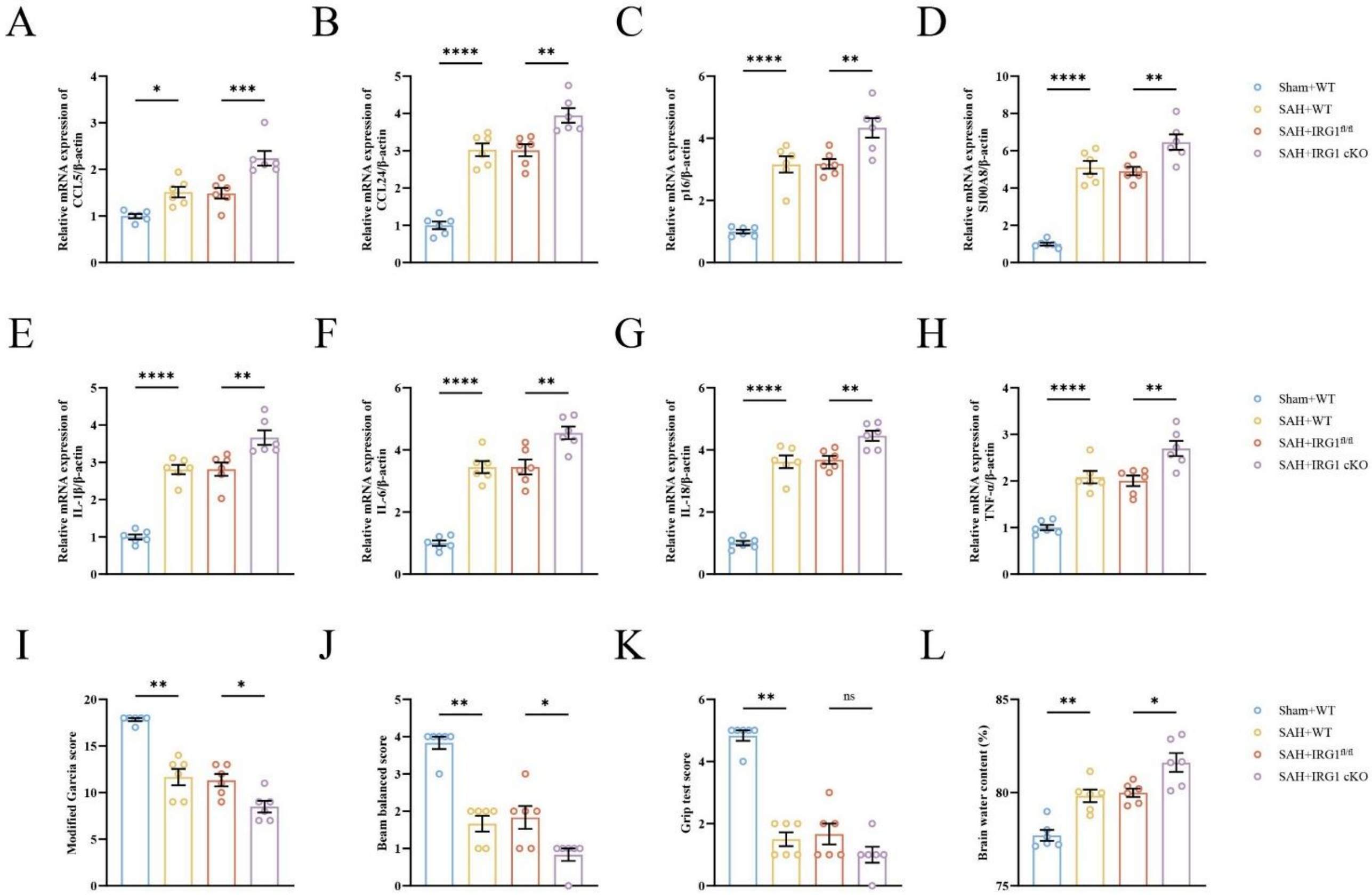

Supplementary Fig. 5. Effects of IRG1 deficiency on microglial senescence-like transition, inflammation, and neurological function after SAH in female mice. A-H. qPCR to evaluate CCL5, CCL24, p16, S100A8, IL-1β, IL-6, IL-18, and TNF-α expressions in mice of Sham+WT, SAH+WT, SAH+IRG1$^{fl/fl}$, and SAH+IRG1 cKO groups (n=6 mice per group). **I-K.** Modified Garcia score, beam balance score, and grip test score to evaluate short-term neurological function (n=6 mice per group). **L.** Measurement of brain water content (n=6 mice per group). Relative mRNA levels were calculated using the $2^{-\Delta\Delta Ct}$ method. All behavioral assessments were performed by two investigators blinded to the experimental groups. Data are presented as mean ± SEM, normalized to the first group, and analyzed by One-way ANOVA (**A-H**, **L**) or Kruskal-Wallis H test (**I-K**).

© 2026. Zhang Z et al. Published online at http://www.aginganddisease.org/EN/10.14336/AD.2025.1527

SUPPLEMENTARY DATA

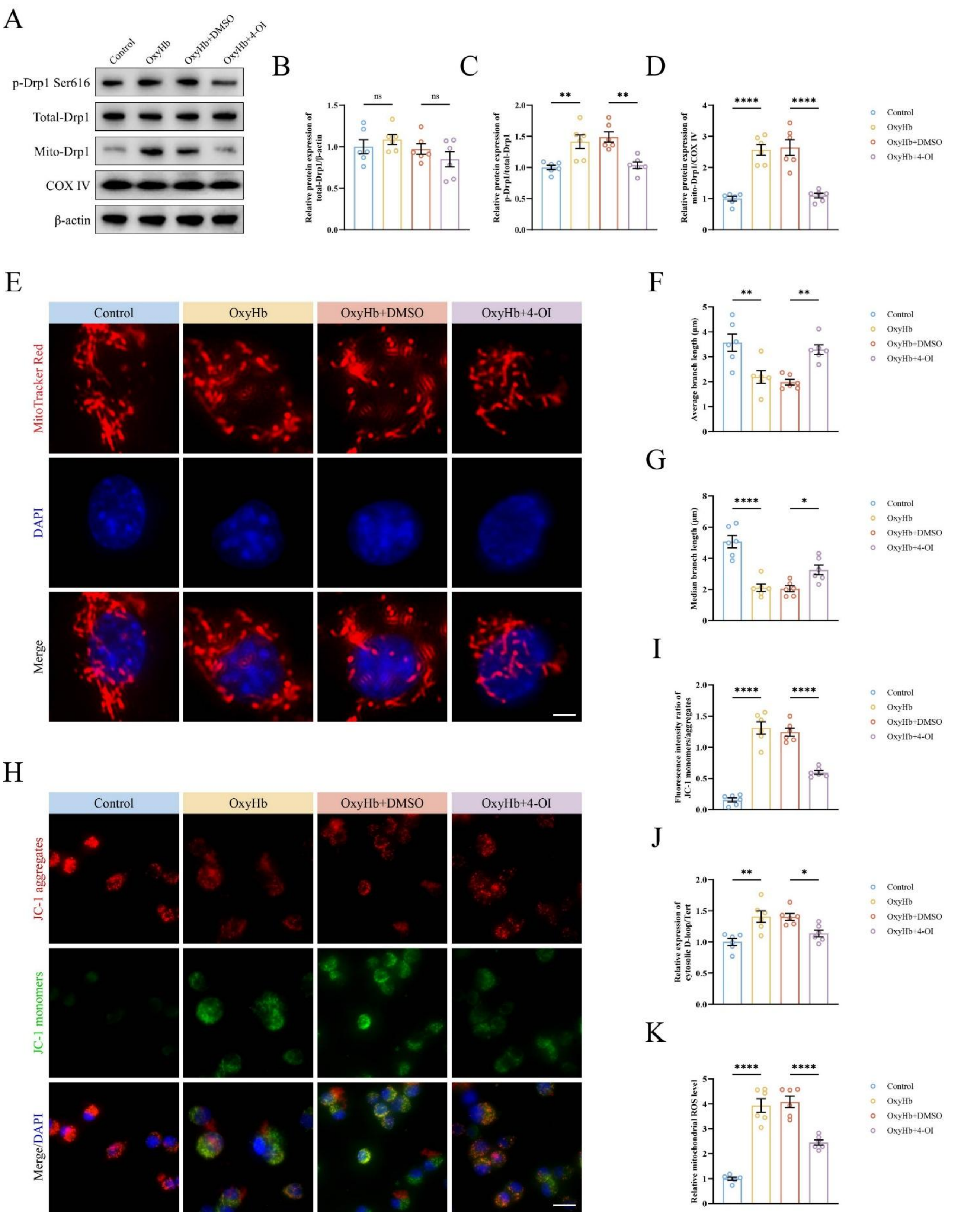

Supplementary Fig. 6. 4-OI inhibits Drp1-dependent mitochondrial fission and improves mitochondrial function in OxyHb-stimulated microglia. A-D. Western blot to evaluate the phosphorylation activation and mitochondrial translocation of Drp1 in BV2 cells treated with Control, OxyHb, OxyHb+DMSO, and OxyHb+4-OI (n=6 independent biological replicates

© 2026. Zhang Z et al. Published online at http://www.aginganddisease.org/EN/10.14336/AD.2025.1527

per group). **E-G.** Mito-Tracker Red CMXRos fluorescent probe to evaluate mitochondrial mean and median branch lengths (scale bar=5μm; n=6 independent biological replicates per group). Mitochondrial morphology was analyzed using the ImageJ mitochondrial morphology macro. **H-I.** JC-1 fluorescent probe to evaluate mitochondrial membrane potential level (scale bar=20μm; n=6 independent biological replicates per group). Fluorescence ratios (Green/Red) were calculated to determine membrane potential. **J.** qPCR to evaluate cytoplasmic D-loop level (n=6 independent biological replicates per group). **K.** MitoSOX Red to evaluate mitochondrial ROS level (n=6 independent biological replicates per group). Band intensities were quantified using ImageJ and normalized to the loading control. Image analysis was performed in a blinded manner. Data are presented as mean ± SEM, normalized to the first group, and analyzed by One-way ANOVA (**B-D**, **F-G**, **I-K**).

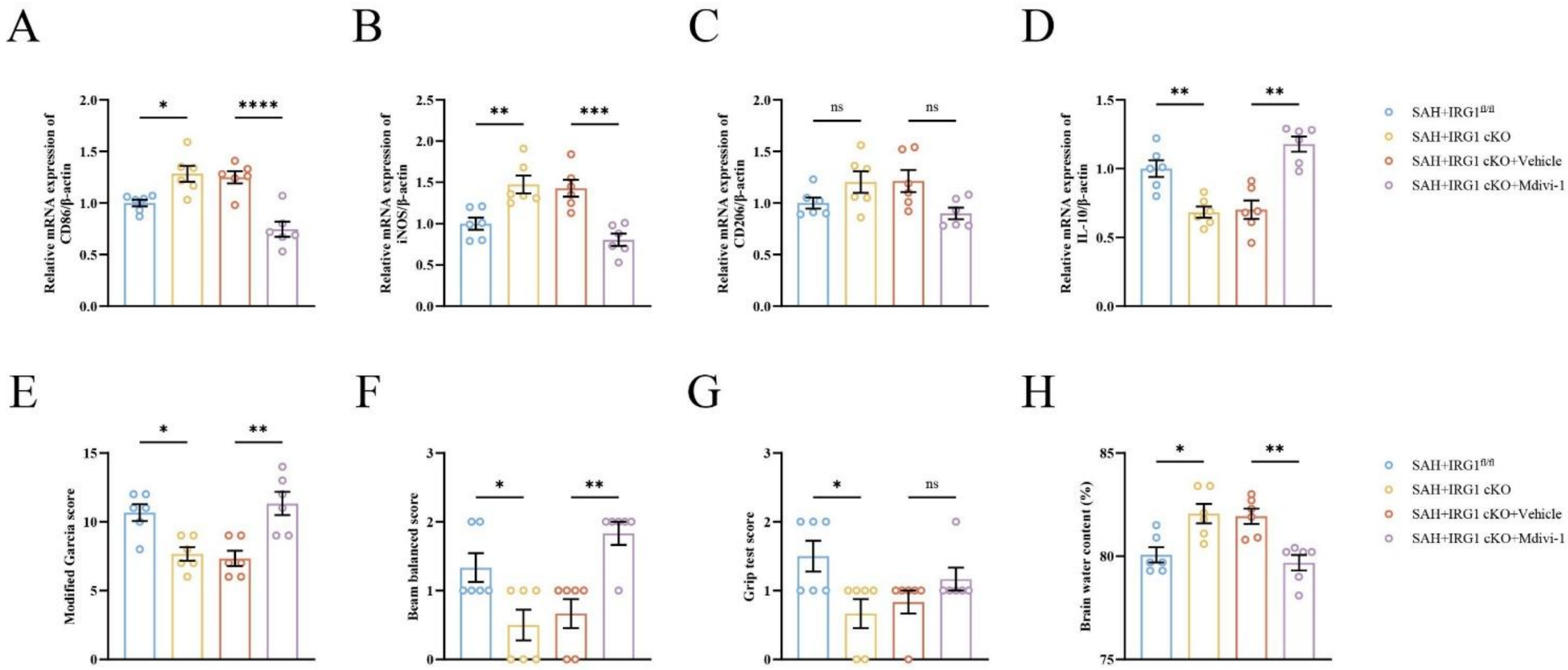

Supplementary Fig. 7. Mdivi-1 rescues the exacerbation of microglial inflammatory activation and neurological deficits caused by IRG1 deficiency. A-D. qPCR to evaluate CD86, iNOS, CD206, and IL-10 expressions in mice of SAH+IRG1$^{fl/fl}$, SAH+IRG1 cKO, SAH+IRG1 cKO+Vehicle, and SAH+IRG1 cKO+Mdivi-1 groups (n=6 mice per group). **E-G.** Short-term neurological function at 24 h post-SAH (n=6 mice per group). Assessments include the modified Garcia score (evaluating 6 parameters: activity, symmetry, extension, climbing, proprioception, and reflex), beam balance, and grip tests. Higher scores indicate superior neurological performance. **H.** Measurement of brain water content (n=6 mice per group). Relative mRNA levels were calculated using the $2^{-\Delta\Delta Ct}$ method. All behavioral assessments were performed by two investigators blinded to the experimental groups. Data are presented as mean ± SEM, normalized to the first group, and analyzed by One-way ANOVA (**A-D**, **H**) or Kruskal-Wallis H test (**E-G**).

SUPPLEMENTARY DATA

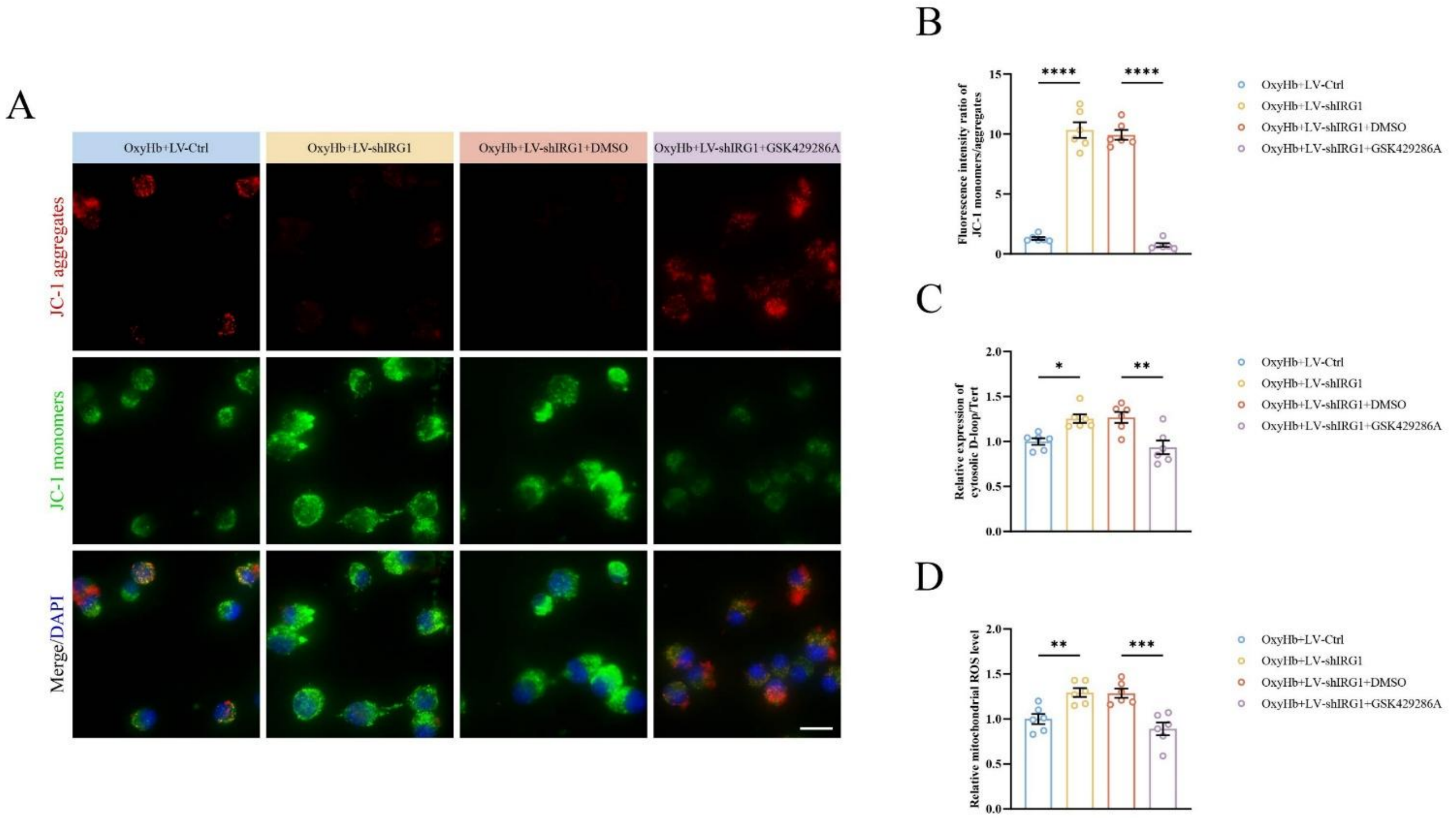

Supplementary Fig. 8. GSK429286A rescues the exacerbated mitochondrial dysfunction caused by IRG1 deficiency. A-B. JC-1 fluorescent probe to evaluate mitochondrial membrane potential level in BV2 cells treated with OxyHb+LV-Ctrl, OxyHb+LV-shIRG1, OxyHb+LV-shIRG1+DMSO, and OxyHb+LV-shIRG1+GSK429286A (scale bar=20μm; n=6 independent biological replicates per group). Fluorescence ratios (Green/Red) were calculated to determine membrane potential. **C.** qPCR to evaluate cytoplasmic D-loop level (n=6 independent biological replicates per group). **D.** MitoSOX Red to evaluate mitochondrial ROS level (n=6 independent biological replicates per group). Image analysis was performed in a blinded manner. Data are presented as mean ± SEM, normalized to the first group, and analyzed by One-way ANOVA (**B-D**).

© 2026. Zhang Z et al. Published online at http://www.aginganddisease.org/EN/10.14336/AD.2025.1527

SUPPLEMENTARY DATA

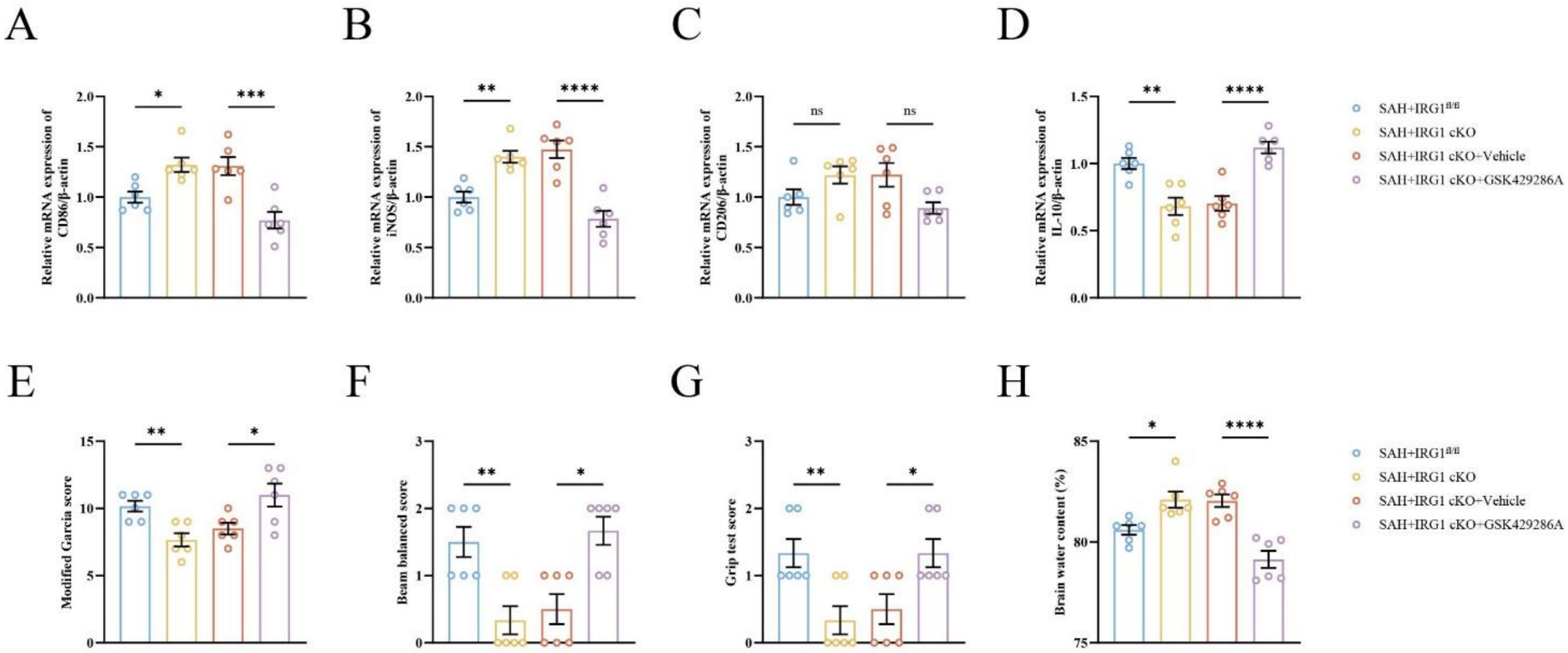

Supplementary Fig. 9. GSK429286A rescues the exacerbation of microglial inflammatory activation and neurological deficits caused by IRG1 deficiency. A-D. qPCR to evaluate CD86, iNOS, CD206, and IL-10 expressions in mice of SAH+IRG1$^{fl/fl}$, SAH+IRG1 cKO, SAH+IRG1 cKO+Vehicle, and SAH+IRG1 cKO+GSK429286A groups (n=6 mice per group). **E-G.** Short-term neurological function at 24 h post-SAH (n=6 mice per group). Assessments include the modified Garcia score (evaluating 6 parameters: activity, symmetry, extension, climbing, proprioception, and reflex), beam balance, and grip tests. Higher scores indicate superior neurological performance. **H.** Measurement of brain water content (n=6 mice per group). Relative mRNA levels were calculated using the $2^{-\Delta\Delta Ct}$ method. All behavioral assessments were performed by two investigators blinded to the experimental groups. Data are presented as mean ± SEM, normalized to the first group, and analyzed by One-way ANOVA (**A-D**, **H**) or Kruskal-Wallis H test (**E-G**).

A

Homo sapiens 80-LMCFSIDSPDSLENIPEKWTPEVKHFCPNVPIILVGNKKDLRNDEHTRRE-131

Mus musculus 80-LMCFSIDSPDSLENIPEKWTPEVKHFCPNVPIILVGNKKDLRNDEHTRRE-131

Rattus norvegicus 80-LMCFSIDSPDSLENIPEKWTPEVKHFCPNVPIILVGNKKDLRNDEHTRRE-131

Pan troglodytes 80-LMCFSIDSPDSLENIPEKWTPEVKHFCPNVPIILVGNKKDLRNDEHTRRE-131

Bos taurus 80-LMCFSIDSPDSLENIPEKWTPEVKHFCPNVPIILVGNKKDLRNDEHTRRE-131

Canis lupus familiaris 80-LMCFSIDSPDSLENIPEKWTPEVKHFCPNVPIILVGNKKDLRNDEHTRRE-131

Sus scrofa 80-LMCFSIDSPDSLENIPEKWTPEVKHFCPNVPIILVGNKKDLRNDEHTRRE-131

Danio rerio 80-LMCFSIDSPDSLENIPEKWTPEVKHFCPNVPIILVGNKKDLRNDEHTRRE-131

Oryctolagus cuniculus 80-LMCFSIDSPDSLENIPEKWTPEVKHFCPNVPIILVGNKKDLRNDEHTRRE-131

Caenorhabditis elegans 80-LMCFSIDSPDSLENIPEKWTPEVRHFCPNVPIILVGNKRDLRSDPQTVRE-131

Supplementary Fig. 10. Analysis of species conservation of the C107 site of RhoA. A. Sequence alignment of the C107 site of RhoA among *Homo sapiens*, *Mus musculus*, *Rattus norvegicus*, *Pan troglodytes*, *Bos taurus*, *Canis lupus familiaris*, *Sus scrofa*, *Danio rerio*, *Oryctolagus cuniculus*, and *Caenorhabditis elegans*.

SUPPLEMENTARY DATA

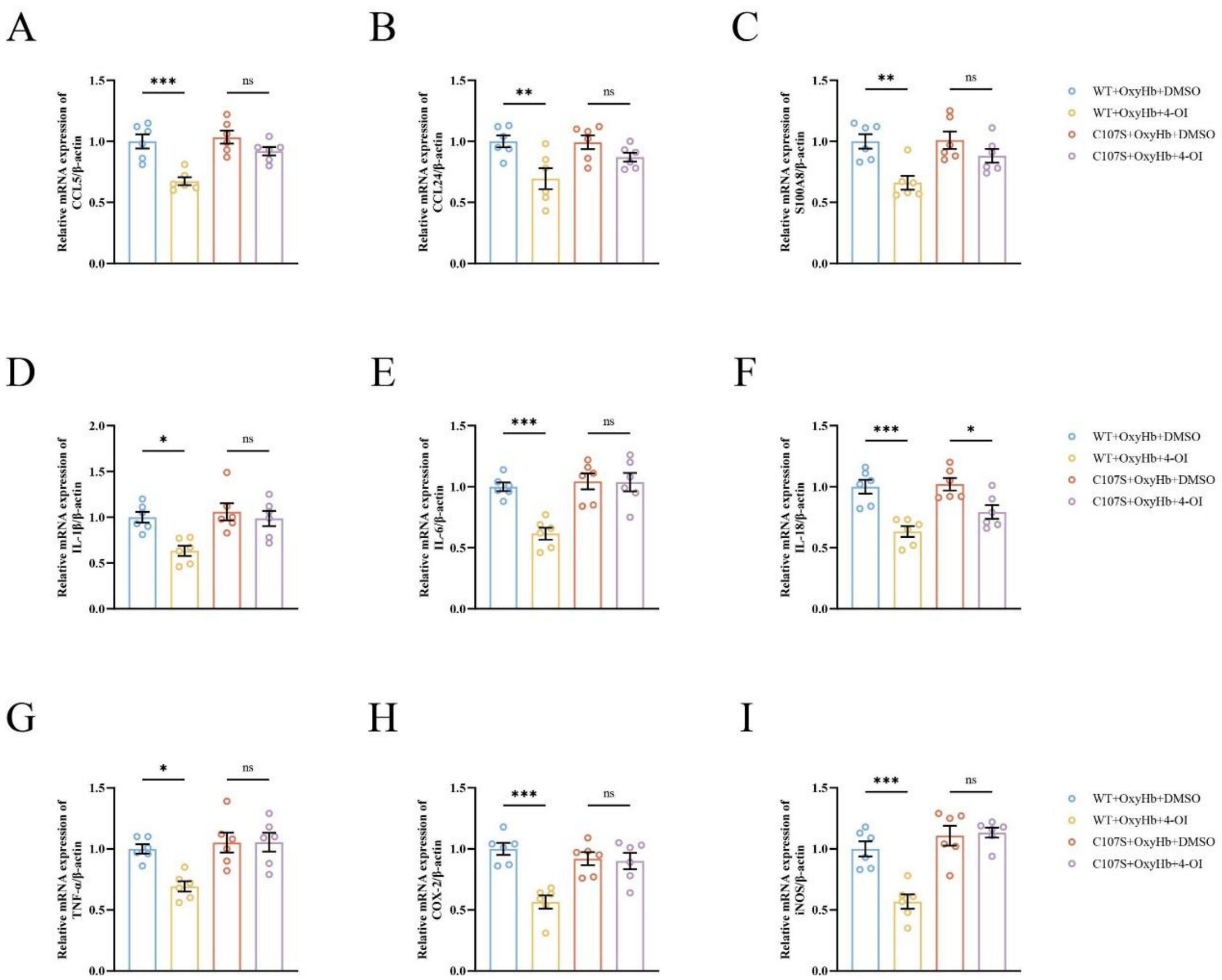

Supplementary Fig. 11. C107S mutation of RhoA reverses the inhibitory effect of 4-OI on senescence-like transition and inflammatory response. A-I. qPCR to evaluate CCL5, CCL24, S100A8, IL-1β, IL-6, IL-18, TNF-α, COX-2, and iNOS expressions in BV2 cells of WT+OxyHb+DMSO, WT+OxyHb+4-OI, C107S+OxyHb+DMSO, and C107S+OxyHb+4-OI groups (n=6 independent biological replicates per group). Relative mRNA levels were calculated using the $2^{-\Delta\Delta Ct}$ method. Data are presented as mean ± SEM, normalized to the first group, and analyzed by One-way ANOVA (**A-I**).

© 2026. Zhang Z et al. Published online at http://www.aginganddisease.org/EN/10.14336/AD.2025.1527

SUPPLEMENTARY DATA

Supplementary Tables

Supplementary Table 1. Primer sequence for qPCR

Gene	5' to 3' Forward Primer Sequence	5' to 3' Reverse Primer Sequence
CCL5	GCAGCAAGTGCTCCAATCTTGC	TCTTCTCTGGGTTGGCACACAC
CCL24	AGCCGGAGGTGTAACTCAGATG	GCTGTGGTTGAAACCCAATGCC
p16	TCAGGTGATGATGATGGGCAACG	TCGAATCTGCACCGTAGTTGAGC
S100A8	TTCGAGGAGTTCCTTGCGATGG	TGTGGCTGTCTTTGTGAGATGCC
IRG1	AGCAACCACGCTGAATGTCTTC	GGCAACATCTGGGACTTAGAACCC
IL-1β	GCAACTGTTCCTGAACTCAACT	ATCTTTTGGGGTCCGTCAACT
IL-6	ACGGCCTTCCCTACTTCACAAG	ACAGGTCTGTTGGGAGTGGTATCC
IL-18	ACAGGCCTGACATCTTCTGCAAC	TGGAAGGTTTGAGGCGGCTTTC
TNF-α	AAGCCTGTAGCCCACGTCGTA	GGCACCACTAGTTGGTTGTCTTTG
COX-2	ACTGCGAGCTAAGAGCTTCAGG	TGCAGTGCTGAGTTCCTTCGTG
iNOS	ATTTGCGGAGATGTGCGCATGG	TGGCCACCAGCTTCTTCAATGTG
CD86	GCCACAGATACTGGAAGAATCCAC	CAGTGCTGCCTGCTAAGTTGTG
CD206	TGAGTCCAGGAACAAGTGACACG	TCGCATGCTCATTCTGCTCGATG
IL-10	TAAGAGCAAGGCAGTGGAGCAG	CATGGCCTTGTAGACACCTTGG
β-actin	GTCGTACCACAGGCATTGTGATGG	GCAATGCCTGGGTACATGGTGG
D-loop	AATCTACCATCCTCCGTGAAACC	TCAGTTTAGCTACCCCCAAGTTTAA
TERT	CTAGCTCATGTGTCAAGACCCTCTT	GCCAGCACGTTTCTCTCGTT

© 2026. Zhang Z et al. Published online at http://www.aginganddisease.org/EN/10.14336/AD.2025.1527

SUPPLEMENTARY DATA

Supplementary Table 2. Antibody information for western blot and immunofluorescence

Antibody	Company	Item number	Application	Dilution
IRG1 antibody	ZEN-BIOSCIENCE	163860	Western Blot	1/1000
p-Drp1 antibody	Abcam	ab314755	Western Blot	1/1000
Drp1 antibody	Bioss	bs-4100R	Western Blot	1/1000
COX IV antibody	ZEN-BIOSCIENCE	250135	Western Blot	1/1000
RhoA antibody	Bioss	bsm-54572R	Western Blot	1/1000
ROCK1 antibody	Bioss	bs-1166R	Western Blot	1/1000
p-ROCK1 antibody	Bioss	bs-4630R	Western Blot	1/1000
ROCK2 antibody	Bioss	bs-1205R	Western Blot	1/1000
β-actin antibody	ZEN-BIOSCIENCE	250132	Western Blot	1/5000
Iba1 antibody	Abcam	ab283319	Immunofluorescence	1/100
CD86 antibody	Proteintech	83213-1-RR	Immunofluorescence	1/200
IRG1 antibody	Bioss	bs-16705R	Immunofluorescence	1/200
